# Supplementary material for: Prolyl hydroxylase-dependent proteolysis enables the orthogonal hypoxia responses in plants
Source: Nat Commun. 2026 Apr 9;17:4834. doi: 10.1038/s41467-026-71366-3 (PMC13223302; doi:10.1038/s41467-026-71366-3)
Supplement: Supplementary file 1 — Supplementary Information [file 41467_2026_71366_MOESM1_ESM.pdf]

Supplementary Information for ‘Prolyl hydroxylase-dependent proteolysis enables the orthogonal hypoxia responses in plants’

Vinay Shukla[1†], Sergio Iacopino[1,2 †], Laura Dalle Carbonare[1], Alessia Del Chiaro[1], Yuming He[1], Mauricio Tronca[1], Thomas P. Keeley[3,4,5], Antonis Papachristodoulou[6], Beatrice Giuntoli[2*], Francesco Licausi[1*]

[1] Department of Biology, University of Oxford, South Parks Road, OX13RB Oxford, United Kingdom.

[2] Department of Biology, University of Pisa, Via Luca Ghini 13, 56126, Pisa, Italy.

[3] Department of Physiology, Anatomy and Genetics, University of Oxford, OX1 3PT Oxford, United Kingdom.

[4] Target Discovery Institute, Nuffield Department of Medicine, University of Oxford, Oxford, United Kingdom.

[5] Ludwig Institute for Cancer Research, Nuffield Department of Medicine, University of Oxford, Oxford, United Kingdom.

[6]Department of Engineering Science, University of Oxford, Parks Road, Oxford OX13PJ, United Kingdom.

[†]these authors contributed equally

*corresponding authors: beatrice.giuntoli@unipi.it , francesco.licausi@biology.ox.ac.uk

Content

Supplementary Figures 1 to 14 2-18

Supplementary Tables 1 to 5 19-23

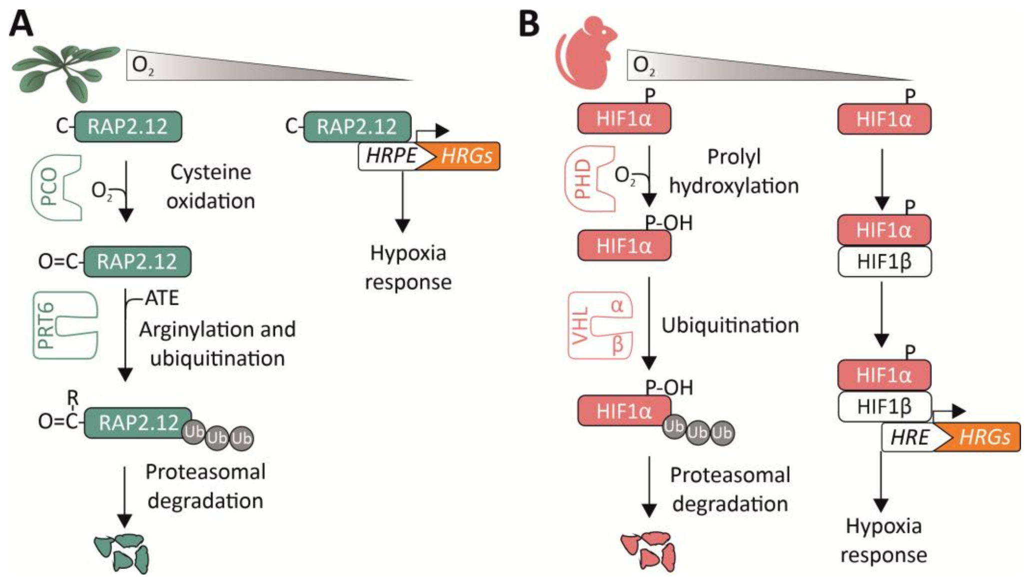

Figure S1: **O_2 sensing in vascular plants and in mammals. A.** Plant O_2 sensing based on the Plant Cysteine Oxidase (PCO)/N-degron pathway. After the removal of the initial Met by Met Aminopeptidases (MAP), the N-terminal Cys residue (C) of ERFVIIs is labelled for proteasomal degradation by the sequential action of PCOs, Arginyl-tRNA transferase (ATE) and the Proteolysis6 (PRT6) E3 ubiquitin ligase. Under hypoxic conditions, ERFVIIs are stabilised and induce the expression of hypoxia-responsive genes. **B.** Animal O_2 sensing based on the PHD/VHL pathway. In the presence of O_2, the α-subunit of the heterodimeric HIF-1 transcription factor is hydroxylated at two proline residues (P) by prolyl hydroxylases (PHDs). This modification marks HIF-1α for proteasomal degradation via the E3 ubiquitin ligase VHL. Under hypoxia, the stabilised HIF-1α forms a dimer with HIF-1β and activates the expression of target genes.

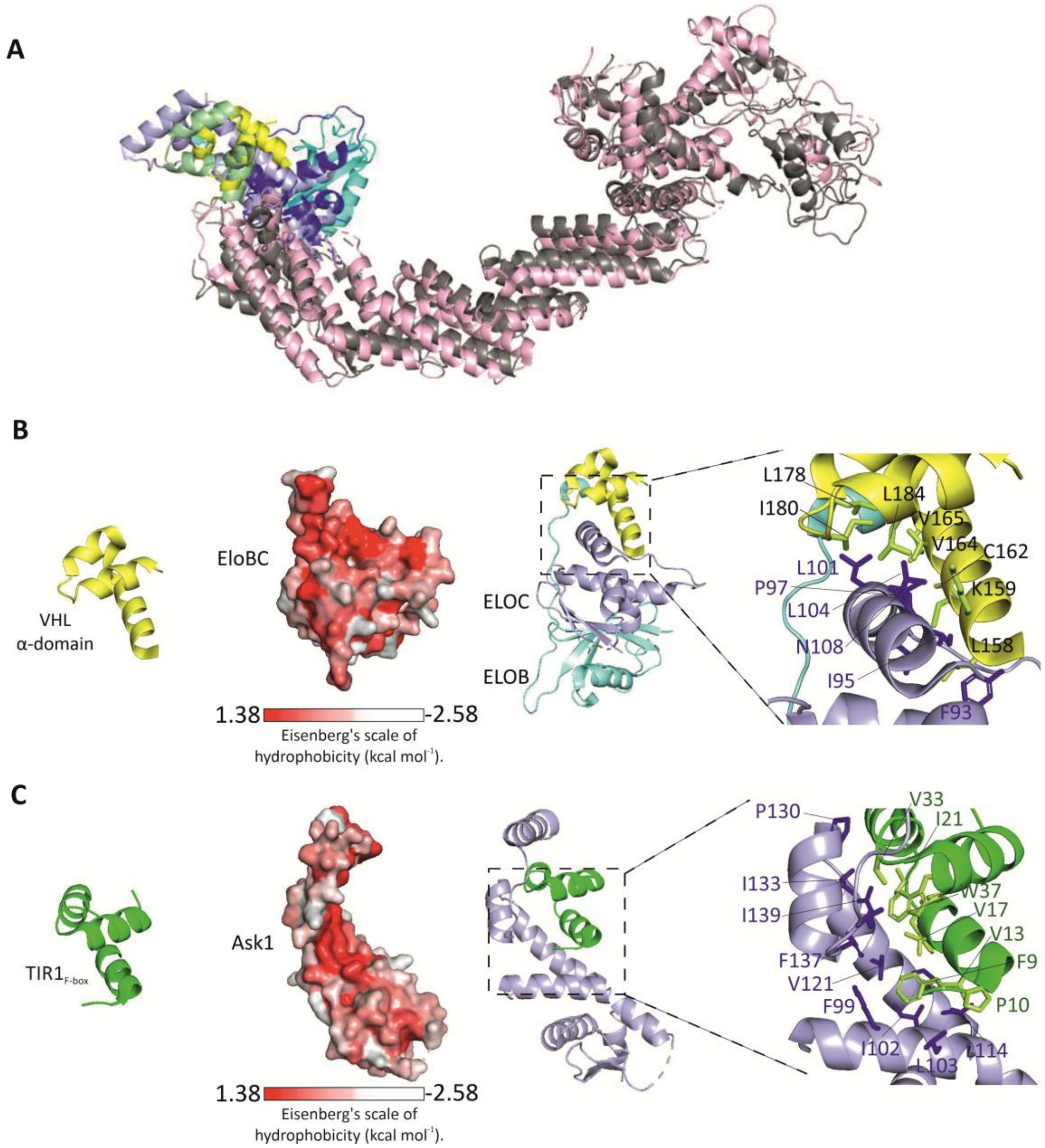

Figure S2. Structural comparison of plant F-box domains and the α-domain of human VHL. A. Overlay of human VBC-CR and Arabidopsis SCF in silico models showing subunits arrangements (human Cullin2 is shown in pink, Arabidopsis Cullin1 in dark gray, and adaptor proteins are shown in the colours used in Fig. 1A). **B.** From left to right: cartoon representation of the VHL α-domain, surface representation of the Elongin B (ELOB) and ELOC heterodimer and cartoon representation of the complex containing the VHL α-domain and ELOB and ELOC. The inset provides a close-up view of the residues involved in the interaction. The VHL α-domain is coloured in yellow, ELOB and ELOC surface was coloured according to the Eisenberg's scale of hydrophobicity using the color_h script in PyMOL. [1]In this scale, red colour indicates hydrophobicity, from least hydrophobic (white) to most hydrophobic (red). In the cartoon representation on the right, ELOB and ELOC are coloured in blue and cyan, respectively. **C.** From left to right: cartoon representation of the F-box domain of Transport Inhibitor Resistant 1 (TIR1), surface representation of the Ask1 protein and cartoon representation of Ask1-TIR1$_{F\text{-}box}$ interaction with a close-up view of the residues involved. The TIR1$_{F\text{-}box}$ is coloured in green, Ask1 surface is coloured according to Eisenberg's scale of hydrophobicity[1] and the Ask1 cartoon is

coloured in blue. TIR1 and ASK1 structures were retrieved from PDB: 2P1O[1]. The structures of VHL, ELOB and ELOC were obtained from PDBs 1VCB[2] and 1LQB[3].

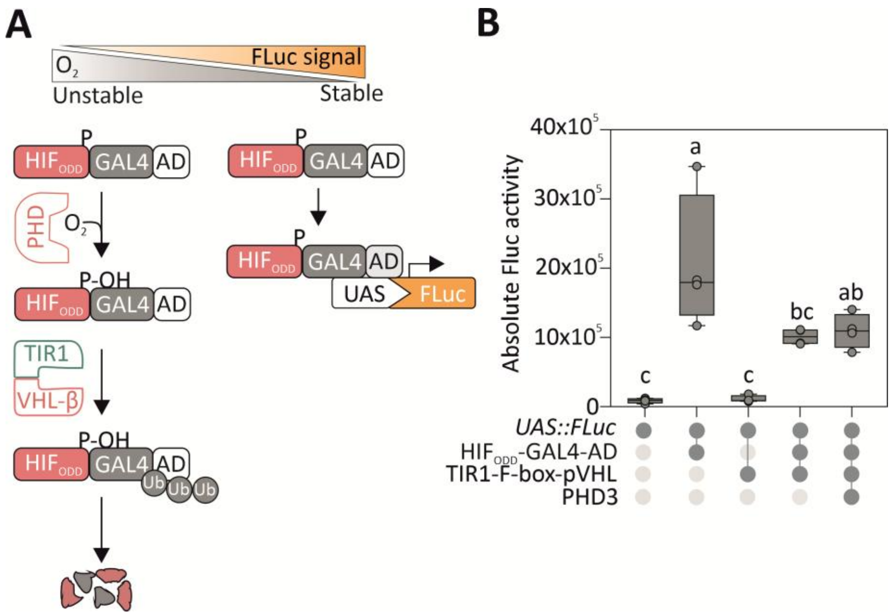

Figure S3. Test of a TIR1-containing hypoxia-inducible O_2 sensor. A. Schematic representation of the synthetic device. The human HIF_{ODD} was equipped with the Gal4 DNA binding domain (DBD) end the Arabidopsis RAP2.12 activation domain (AD), while the VHL β-domains (VHL) was fused in frame with the F-box domain of TIR1. In the presence of O_2, the O2-dependent degradation domain of HIF_{ODD}-GAL4-AD is hydroxylated by PHD3 and therefore recognised by VHL, which mediates its ubiquitination and subsequent proteasomal targeting. At low O_2 levels, instead, HIF_{ODD}-GAL4-AD can bind repeats of the GAL4 upstream activating sequence (4xUAS) in a synthetic promoter and induce the transcription of a firefly luciferase reporter (FLuc). **B.** Sensor output observed in Arabidopsis mesophyll protoplasts after transfection with different combinations of the effector modules HIF_{ODD}-GAL4-AD, $TIR1_{F\text{-}box}$ -VHL and PHD3 (empty circles, effector absent; dark circles, effector present). Letters indicate statistical differences ($p \leq 0.05$) calculated by a two-way ANOVA comparison followed by Tukey's post-test (n=4 independent biological replicates, consisting in independent transfections performed on aliquots of protoplasts isolated from 18 leaves excides from a batch of 6 plants). Data are shown as box plots, where the centre line indicates the median, box bounds indicate the 25th and 75th percentiles, whiskers extend to the minimum and maximum values, and all points represent individual biological replicates.

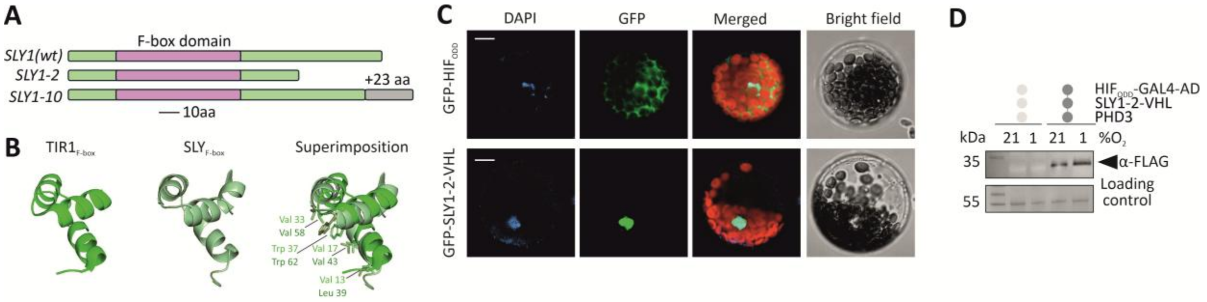

Figure S4. Design of a SLY1-containing hypoxia-inducible O_2 sensor and localization of its effector modules. A. Schematic representation of wild-type SLY1 and the two mutant variants SLY1-2 and SLY1-10. Both variants consist of SLY1 truncations, with an additional 23 aa-long domain at SLY1-10 C-terminal end. **B.** Comparison of the structure of the F-box domain of TIR1 and SLY1 and their superimposition showing the conservation of residues depicted in Supplementary figure 2B. **C.** Subcellular localization of the GFP-SLY1-2-VHL and GFP-HIF_{ODD} constructs imaged in isolated Arabidopsis mesophyll protoplasts. DAPI staining marks the nuclei. In the merged image, the red colour is associated with chloroplast auto-fluorescence. Scale bar = 10µm. D. Immunodetection of HIF_{ODD}-GAL4-AD using an anti-FLAG antibody in the same experimental setup as **Fig. 1F**. The experiment was repeated independently 2 times with similar results.

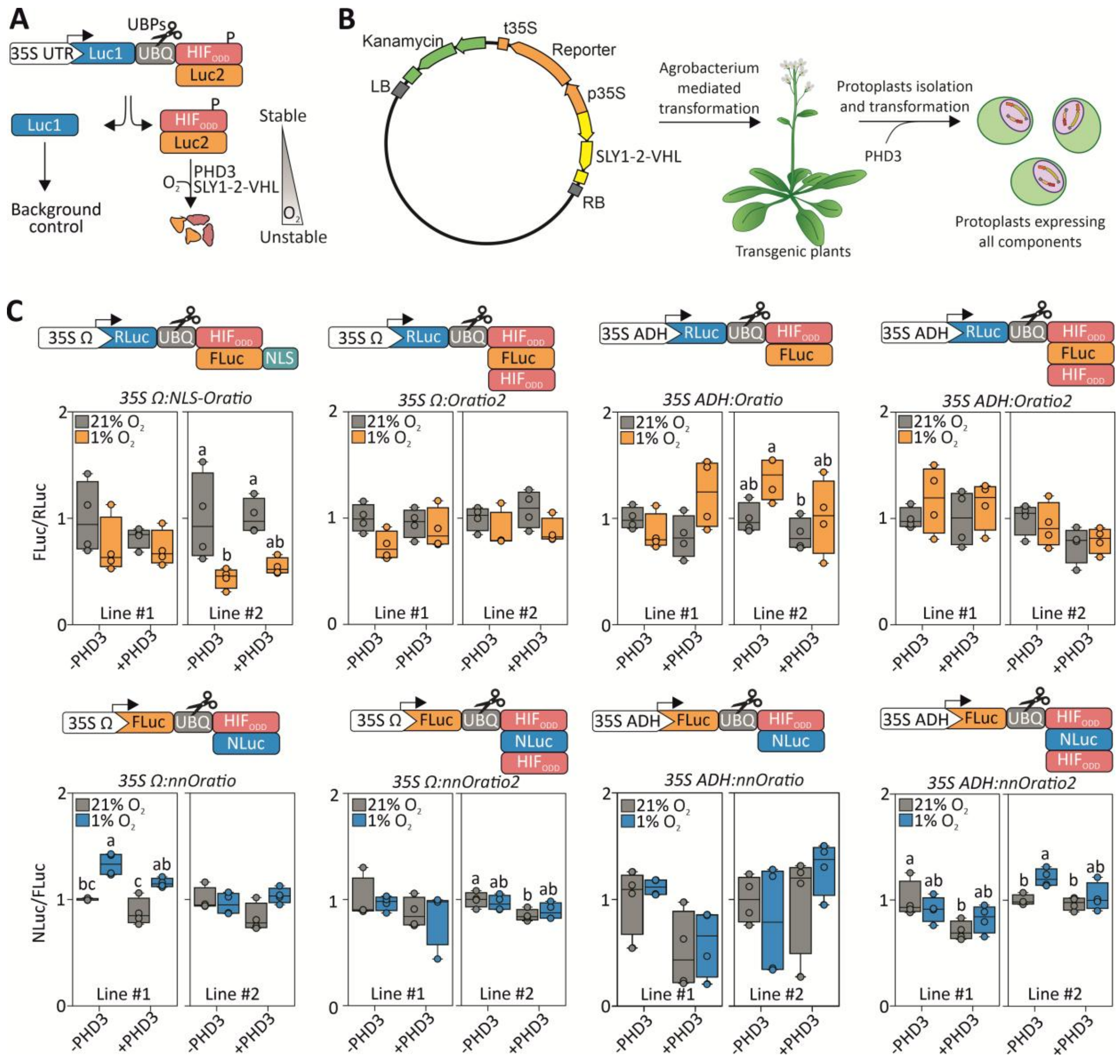

Figure S5. Development and performance of a ratio-metric O_2 sensors in plants. A. A polyprotein consisting of luciferase 1 (Luc1), UBQ10 (UBQ) monomer, and HIF_{ODD} fused to a different luciferase (Luc2) is expressed in plant cells. Endogenous ubiquitin proteases (UBPs) co-translationally cleave the polypeptide at the c-terminus of UBQ10. The resulting LUC1-UBQ serves as a normalization reference while HIF_{ODD}-LUC2 is used to monitor O_2 levels. **B.** Strategy used to test variants of the synthetic O_2 reporter in protoplasts. Two stable transgenic lines were generated for each reporter and SLY1-2-VHL construct. Protoplasts were obtained from each line to test the effect of PHD3 expression on reporter activity under aerobic (21% O_2) and hypoxic (1% O_2) conditions. Such treatments were applied 12 h after protoplast transection and lasted 12 h. **C.** Relative output of different reporter variants tested using the strategy described in (B). FLUC2/FLUC1 ratio in the absence of PHD3 was set to 1 FLuc, firefly luciferase; RLuc, renilla luciferase; NLuc, nanoluciferase. 35S Ω, CamV 35S promoter with omega leader; 35S ADH, CamV 35S promoter with 5'-UTR sequence from the Arabidopsis *ADH1* gene; NLS, nuclear localization signal from *S. cerevisiae* Gal4. Letters indicate statistical differences ($p \leq 0.05$) determined by two-way ANOVA followed by Tukey's post-hoc test (n = 4 independent biological replicates, consisting in independent transfections performed on aliquots of protoplasts isolated from 18 leaves excides from a batch of 6 plants). Data are shown as box plots, where the centre

line indicates the median, box bounds indicate the 25th and 75th percentiles, whiskers extend to the minimum and maximum values, and all points represent individual biological replicates.

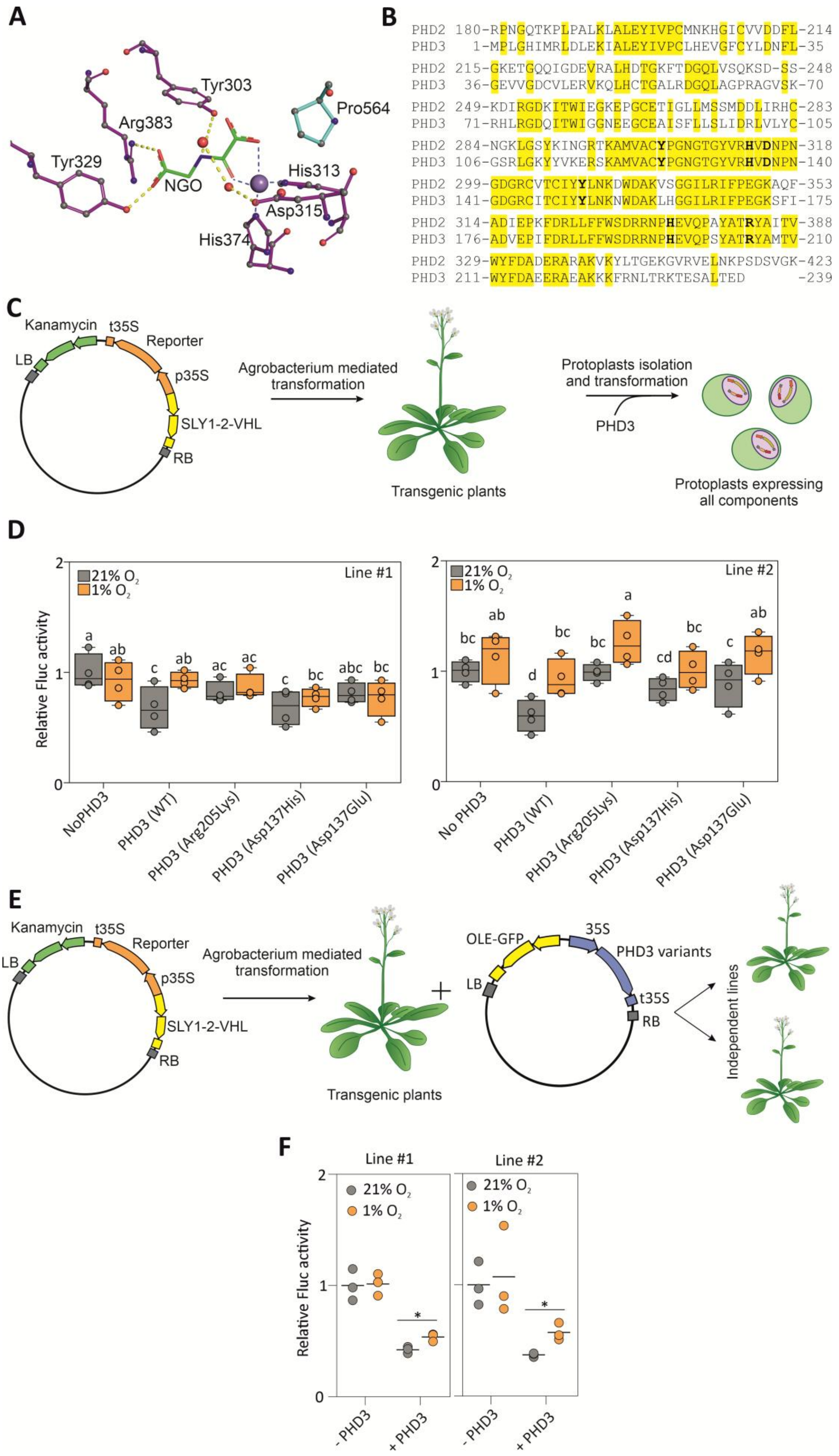
A
Tyr303
Pro564
Arg383
Tyr329
His313
NGO
Asp315
His374
B
PHD2 180-RPNGQTKPLPALKLALEYIVPCMNKHGICVVDDFL-214
PHD3 1-MPLGHIMRLDLEKIALEYIVPCLHEVGFCYLDNFL-35
PHD2 215-GKETGQQIGDEVRALHDTGKFTDGQLVSQKSD-SS-248
PHD3 36-GEVVGDCVLERVKQLHCTGALRDGQLAGPRAGVSK-70
PHD2 249-KDIRGDKITWIEGKEPGCETIGLLMSSMDDLIRHC-283
PHD3 71-RHLRGDQITWIGGNEEGCEAISFLLSLIDRLVLYC-105
PHD2 284-NGKLGSYKINGRTKAMVACYPGNGTGYVRHVDNPN-318
PHD3 106-GSRLGKYYVKERSKAMVACYPGNGTGYVRHVDNPN-140
PHD2 299-GDGRCVTCIYYLNKDWDAKVSGGILRIFPEGKAQF-353
PHD3 141-GDGRCITCIYYLNKNWDAKLHGGILRIFPEGKSFI-175
PHD2 314-ADIEPKFDRLLFFWSDRRNPHEVQPAYATRYAITV-388
PHD3 176-ADVEPIFDRLLFFWSDRRNPHEVQPSYATRYAMTV-210
PHD2 329-WYFDADERARAKVKYLTGEKGVRVELNKPSDSVGK-423
PHD3 211-WYFDAEERAEAKKKFRNLTRKTESALTED -239
C
Kanamycin
t35S
Reporter
LB
p35S
SLY1-2-VHL
RB
Agrobacterium mediated transformation
Transgenic plants
Protoplasts isolation and transformation
PHD3
Protoplasts expressing all components
D
Line #1
Line #2
21% O2
1% O2
Relative Fluc activity
NoPHD3
No PHD3
PHD3 (WT)
PHD3 (Arg205Lys)
PHD3 (Asp137His)
PHD3 (Asp137Glu)
E
Kanamycin
t35S
Reporter
LB
p35S
SLY1-2-VHL
RB
Agrobacterium mediated transformation
Transgenic plants
OLE-GFP
35S
PHD3 variants
t35S
LB
RB
Independent lines
F
Line #1
Line #2
21% O2
1% O2
Relative Fluc activity
- PHD3
+ PHD3

Figure S6. Generation and testing of PHD3 variants. A. Catalytic site of PHD2 human protein extracted from the PDB file 2G19[4] and readapted from (Tarhonskaya et al., 2014). **B.** Amino acid sequence alignment of PHD2 and PHD3. The conserved residues are highlighted in yellow. Bold font indicates residues represented in A. **C.** Strategy used to test expression of PHD3 variants in protoplasts stably expressing O_2ratio and SLY1-2-VHL. **D.** Sensor output in mesophyll protoplasts isolated from O_2ratio and SLY1-2-VHL expressing Arabidopsis plants and transformed with PHD3 expression plasmids. 12 h after transformation, protoplasts were either subjected to 6h of hypoxia (1% O_2) or normoxia (21% O_2). The FLuc and RLuc ratio in the absence of PHD3 was set to 1. Letters indicate statistical differences ($p \leq 0.05$) calculated by a two-way ANOVA comparison followed by Tukey's post-test (n=4 independent biological replicates, consisting in independent transfections performed on aliquots of protoplasts isolated from 18 leaves excides from a batch of 6 plants. Data are shown as box plots, where the centre line indicates the median, box bounds indicate the 25th and 75th percentiles, whiskers extend to the minimum and maximum values, and all points represent individual biological replicates.. **E.** Strategy used to test efficiency of PHD3 variants in plants stably expressing O_2ratio, SLY1-2-VHL and different PHD3 variants. **F.** Sensor output in stable transgenic plants expressing O_2ratio module and SLY1-2-VHL super-transformed with binary plasmid containing PHD3 variants. One-week-old seedlings were either subjected to 6 h of hypoxia (1% O_2) or normoxia (21% O_2). The FLuc/RLuc ratio in the absence of PHD3 was set to 1. Letters indicate statistical differences ($p \leq 0.05$) calculated by a two-way ANOVA comparison followed by Tukey's post-test (n=3 independent biological replicates, consisting in independent transfections performed on aliquots of protoplasts isolated from 12 leaves excides from a batch of 4 plants. Data are shown as dot plots, where the centre line indicates the mean and all points represent individual biological replicates.

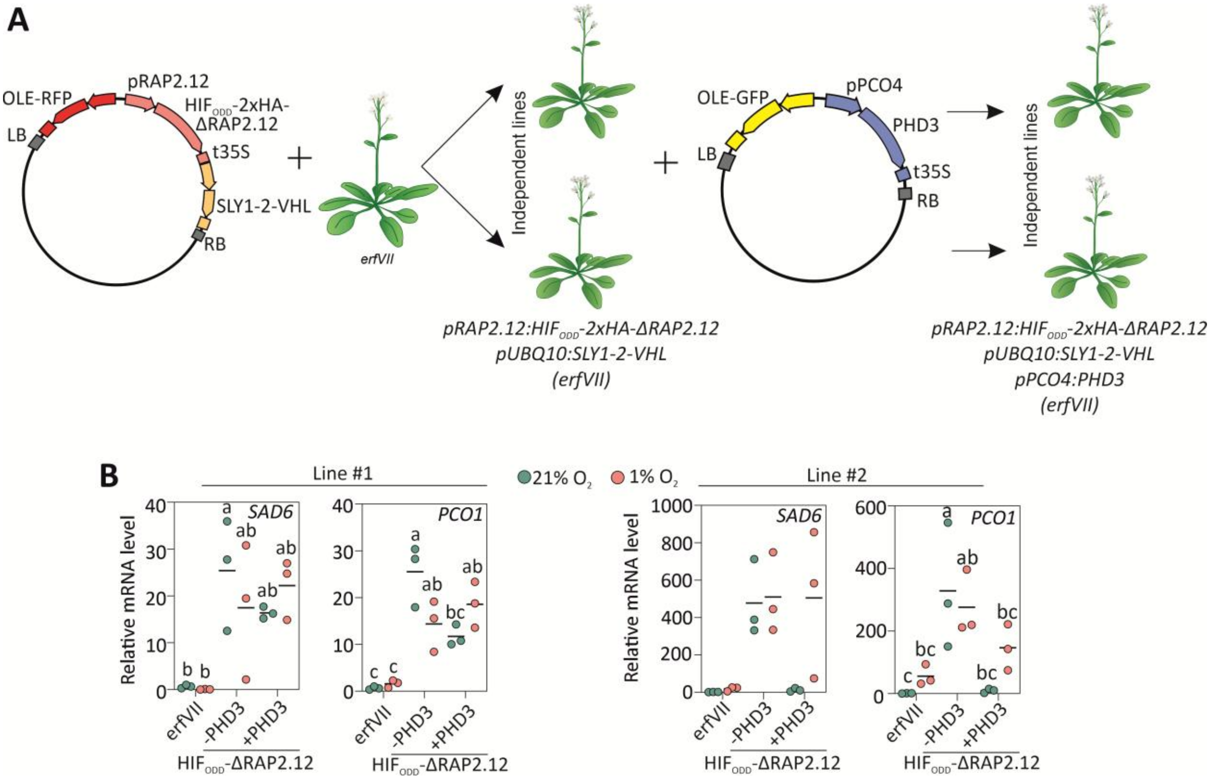

Figure S7. Complementation of the *erfVII* mutant with the chimeric O_2 sensing system. A. Two independent transgenic lines expressing HIF_{ODD}-ΔRAP2.12 and SLY1-2-VHL in the *erfVII* background were super-transformed with a binary vector containing *pPCO4:PHD3* to complete the O_2 sensing system. **B.** Two independent lines expressing HIF_{ODD}-ΔRAP2.12, SLY1-2-VHL and PHD3 were tested for PHD- and O_2-dependent expression of hypoxia marker genes *SAD6* and *PCO1*. Seven-day-old Arabidopsis seedlings were exposed to 21% or 1% O_2 for 2 hours. Letters indicate statistical significance evaluated by a two-way ANOVA followed by the Tukey's post-hoc test for multiple comparisons (n=3 independent biological replicates, consisting in independent transfections performed on aliquots of protoplasts isolated from 18 leaves excides from a batch of 6 plants). Data are shown as dot plots, where the centre line indicates the mean and all points represent individual biological replicates.

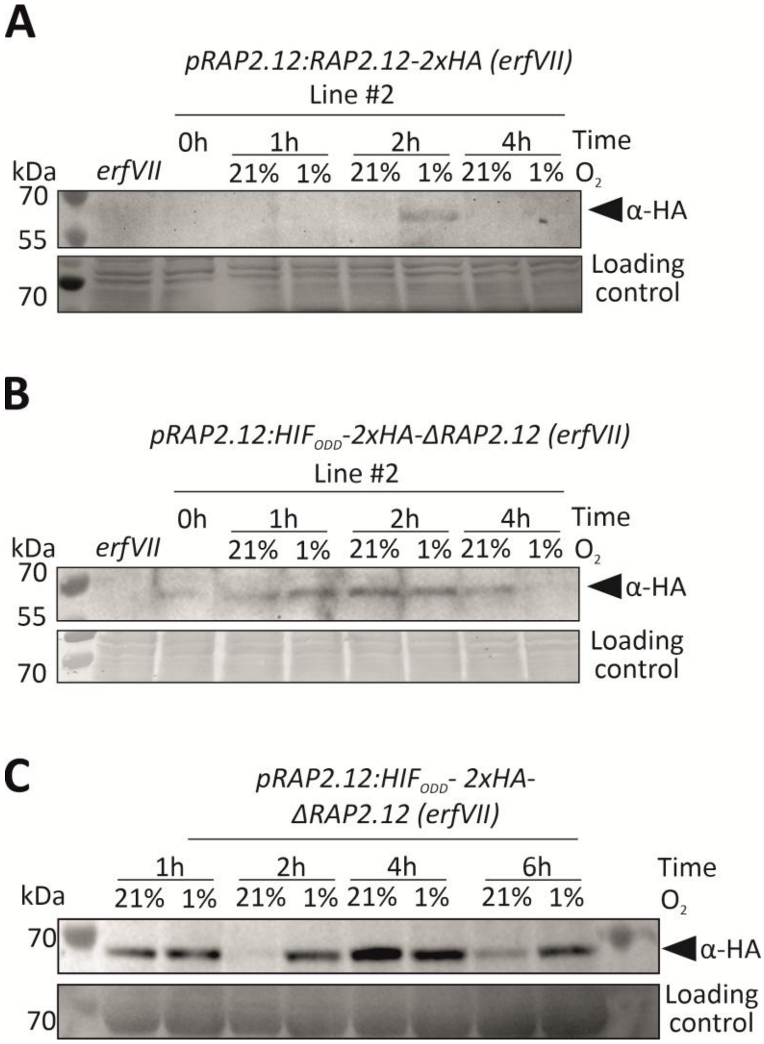

Figure S8. Immunodetection of RAP2.12 in the *erfVII* background complemented with the original and chimeric oxygen sensing systems. A. Immunodetection of RAP2.12-2xHA (line #2) under the regulation of the PCO degradation pathway following a treatment with 1% O_2 for 0 to 4 hours. The untransformed *erfVII* genotype (1% O_2, 2h) is included as a negative control. **B.** Immunodetection of HIF_{ODD}-ΔRAP2.12-2xHA (line #2) under aerobic conditions or after treatment with 1% O_2 for 0 to 4 hours, with the *erfVII* genotype (1% O_2, 2h) included as a negative control. Ponceau staining of total loaded proteins serves as the loading control for Western blots. **C**. Immunodetection of HIF_{ODD}-ΔRAP2.12-2xHA (line #2) under aerobic conditions or after treatment with 1% O_2 for 1, 2, 4 and 6 hours. Ponceau staining of total loaded proteins serves as the loading control for Western blots. These experiments were repeated independently with 2 lines with similar results.

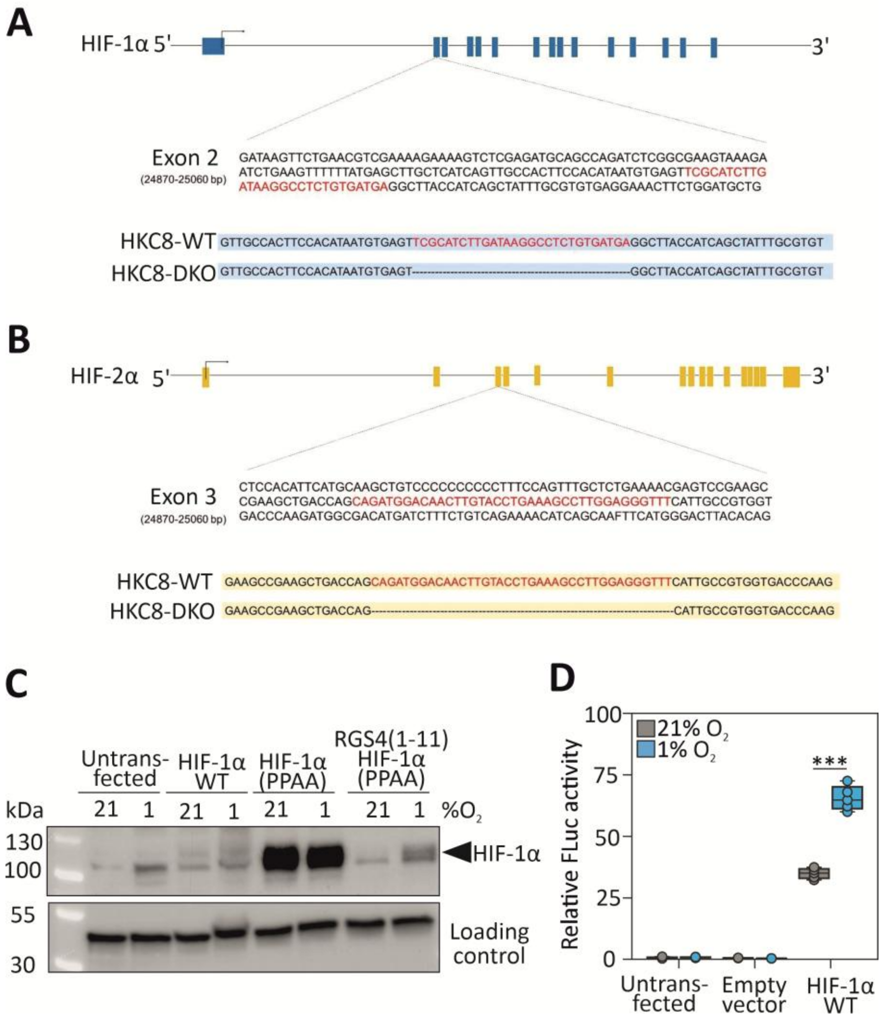

Figure S9. Assessment of HIF1α and HIF2α deletion in HKC-8 DKO cells. A-B. Schematic diagram of HIF1α (**A**) and HIF2α (**B**) gene structure, with a focus on the exonic sequence targeted by the CRISPR/Cas9-mediated genetic editing. The highlighted sequence corresponds to the sequenced genomic DNA of wt and DKO HKC-8 cells. **C.** Immunodetection of HIF-1α in DKO cells transiently transfected with plasmids coding for wild type HIF-1α, HIF-1α(PPAA) and $RGS4_{1-11}$-HIF-1α(PPAA) and treated with 21% or 1% O_2 for 16 h. Beta-actin is shown for equal loading. The experiment was repeated independently 2 times with similar results. **D.** Fluc/Rluc signal at 21% O_2 and 1% O_2 (16 h) of DKO cells transiently transfected with empty or HIF-1α bearing plasmid. Statistical significance of differences was assessed by two-way ANOVA followed by Tukey's post-test ($p<0.05$, n = 5). Data are shown as box plots, where the centre line indicates the median, box bounds indicate the 25th and 75th percentiles, whiskers extend to the minimum and maximum values, and all points represent individual biological replicates.

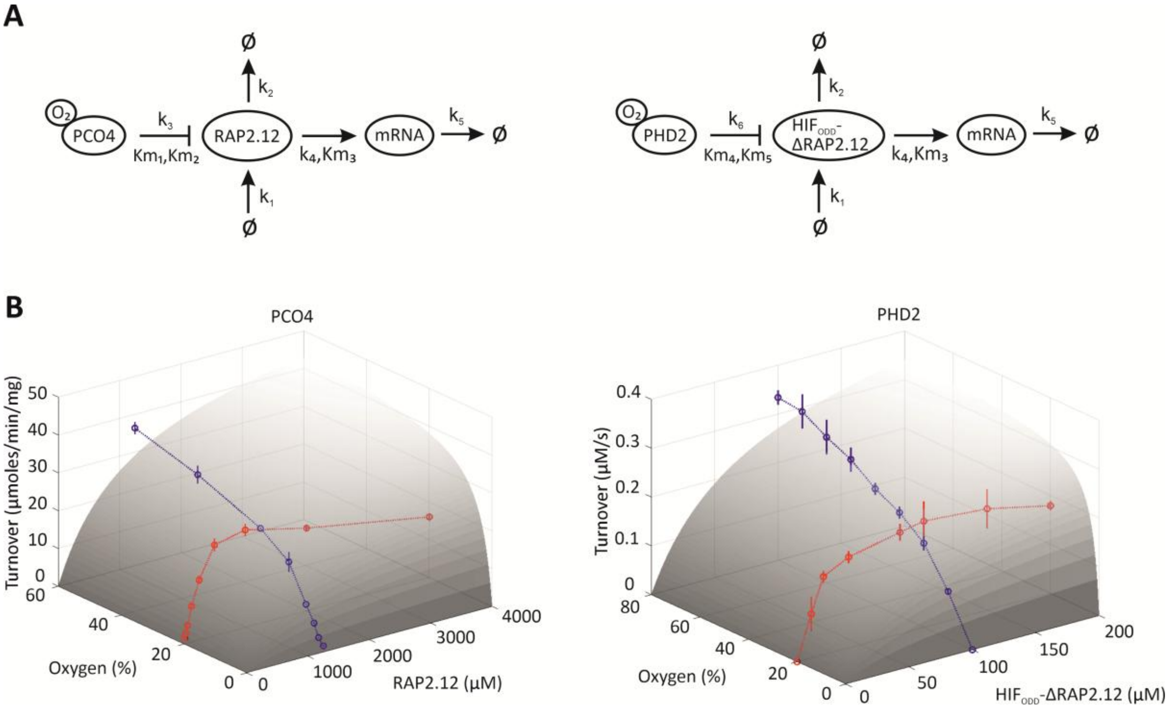

Figure S10. Mathematical modelling of RAP2.12 abundance and target gene expression depending on O_2 levels. A. Schematic representation of models for the native (left) and the synthetic (right) O_2 sensing system tested in plants. All model elements including RAP2.12, HIF-RAP2.12, PCO4, PHD, O_2 and *HRG* mRNA are circled, and 'Ø' symbol stands for 'null'. **B.** 3D data visualization and nonlinear fitting for RAP2.12/PCO4 and HIF-RAP2.12/PHD2 kinetics. The 3D surface plot (grey) shows predicted RAP2.12 turnover rates as a function of oxygen and substrate concentrations. Enzyme kinetics at different substrate concentrations are shown in red. Enzyme kinetics at different oxygen concentrations are shown in blue. Kinetic data are extracted from [5,6]. Error bars represent SE.

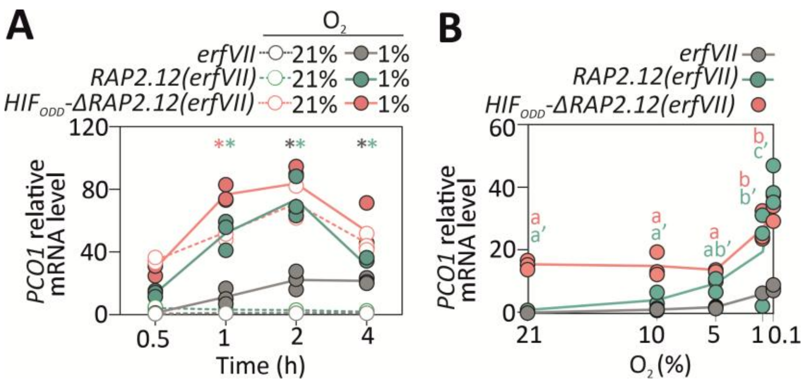

Figure S11. PCO1 gene expression in the *erfVII* background complemented with the original and chimeric oxygen sensing systems. A. Expression of the hypoxia marker gene *PCO1* in 7-day-old Arabidopsis seedlings grown under normoxia and then subjected to 21% or 1% O_2 for 0.5 to 4 hours. Statistical significance of gene expression relative to controls is denoted by asterisks: grey for *erfVII*, pink for HIF-ΔRAP2.12-2xHA and green for RAP2.12-2xHA (n = 3). **B.** Dynamics of *PCO1* marker gene expression in response to varying O_2 levels in plants expressing RAP2.12 under the control of the PCO or PHD pathways in an *erfVII* background. Letters indicate statistical significance across genotypes, analysed by one-way ANOVA for different O_2 percentages, followed by Tukey's post-hoc test against the normoxic samples (n = 3).

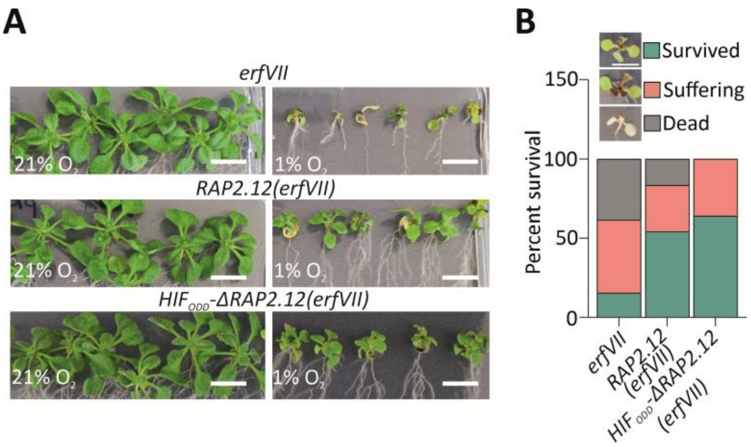

Figure S12. Hypoxia tolerance assay of *erfVII* mutants and complemented lines expressing *RAP2.12-HA* or *HIF$_{ODD}$-ΔRAP2.12-HA*. **A.** Seven-day-old seedlings were grown on agar plates and exposed to 1% O_2 for seven days, followed by a seven-day recovery period. Images were taken at the end of the recovery period. The experiment was repeated independently 2 times with similar results. **B.** Plant tolerance after hypoxia (n ≥ 36). Inset images illustrate representative status for categorisation and quantification.

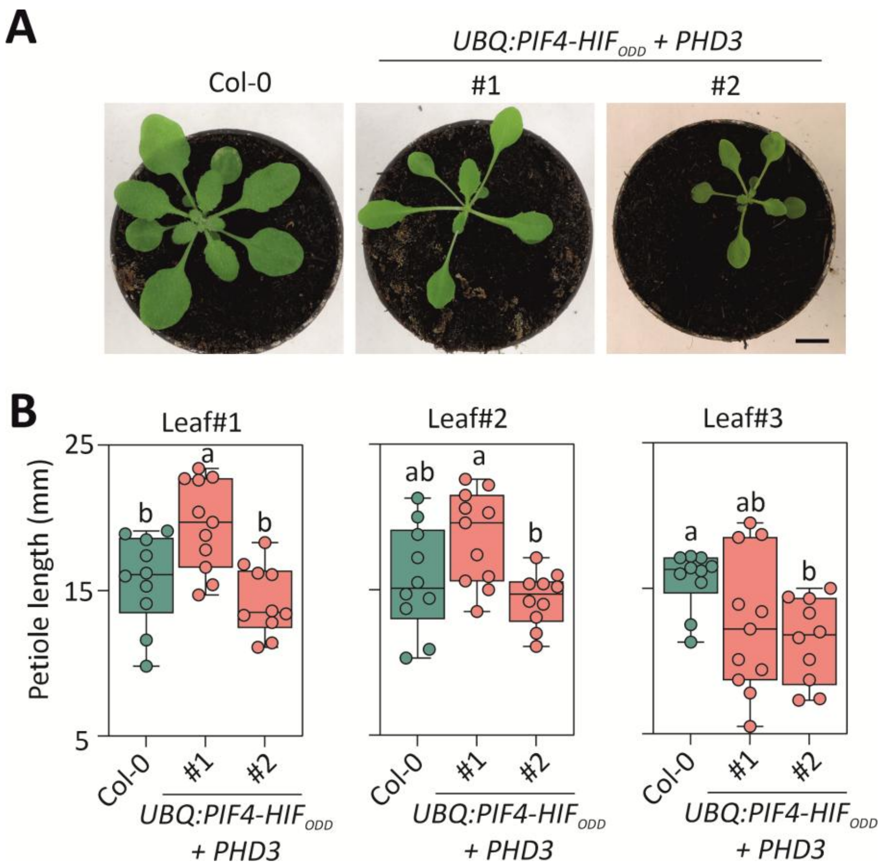

Figure S13. Characterisation of Arabidopsis expressing PIF4-HIF$_{ODD}$ under control of the *UBQ10* promoter. A. Phenotypes of wild-type Col-0 plants and two independently transformed plants expressing *PIF4-HIF$_{ODD}$* under the control of the constitutive UBQ10 promoter (Scale = 1 cm). **B.** Quantification of petiole length of leaves 1 to 3 in *PIF4-HIF$_{ODD}$* plants. Statistical significance, as determined using two-way ANOVA followed by Tukey's post-test ($p \leq 0.05$) is indicated by letters. Data are mean individual petiole length, per genotype and treatment, measured from different plants. Data are shown as box plots, where the centre line indicates the median, box bounds indicate the 25th and 75th percentiles, whiskers extend to the minimum and maximum values, and all points represent individual biological replicates (n=10).

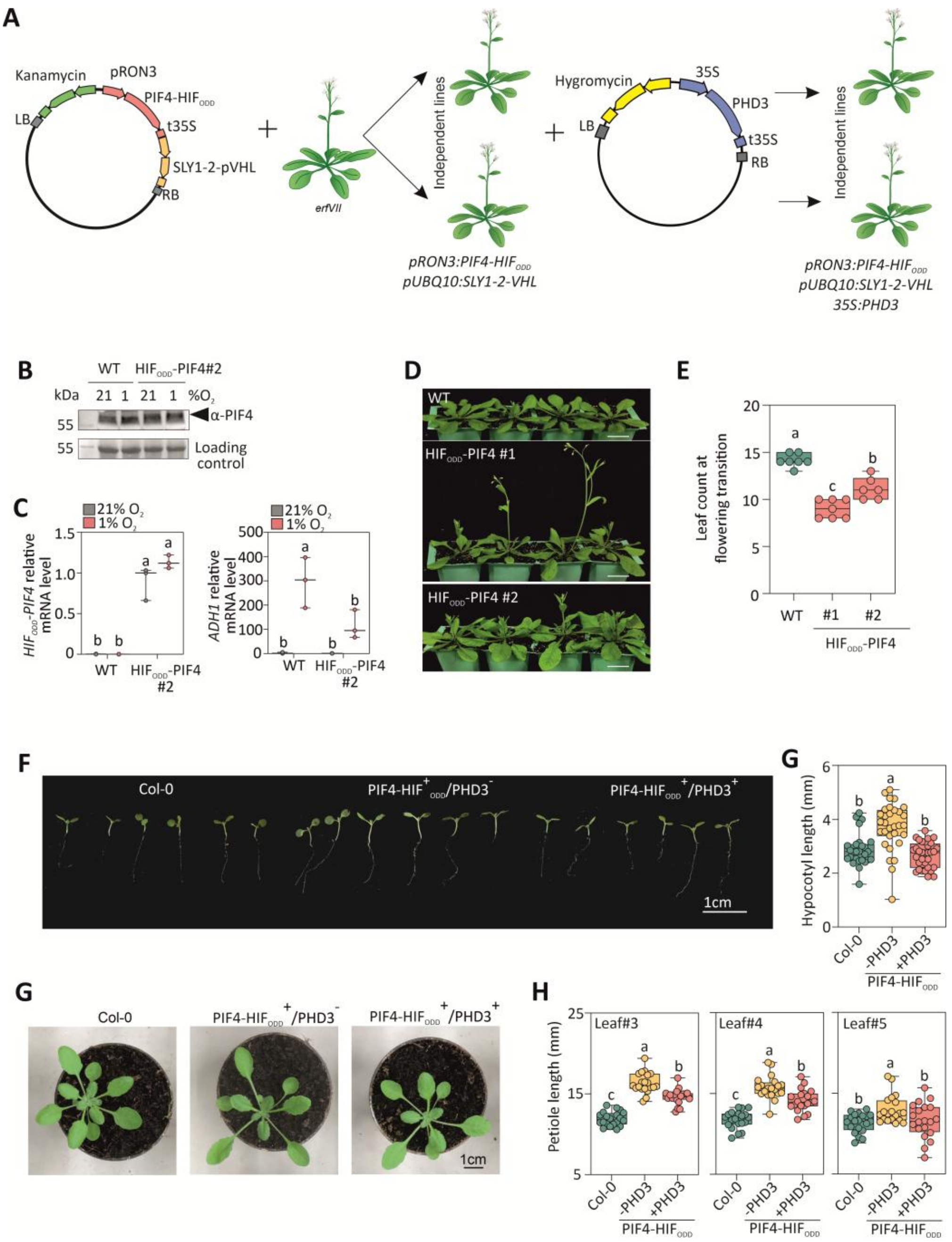

Figure S14. The PIF4-HIF$_{ODD}$ chimera regulates organ elongation in an O_2 dependent manner. **A.** Implementation of flooding-conditional elongation was achieved in two steps. First, plants were transformed with a construct expressing *HIF$_{ODD}$-PIF4* and *SLY1-2-VHL*. Subsequently, two independent lines were super-transformed with a binary vector containing *35S:PHD3* to control PIF4-HIF$_{ODD}$ stability. **B.** Immunodetection of *HIF$_{ODD}$-PIF4* in 7-day old seedlings following a treatment with 21% or 1% O_2 for 6 hours. Ponceau staining of total proteins is shown as the loading control. The Col-0 background is shown as a negative control. **C.** Relative mRNA levels of *HIF$_{ODD}$-PIF4* and *ADH1* in 7-day old seedlings following a treatment with 21% or 1% O_2 for 6 hours. The Col-0 background is shown as a negative control. **D.** Accelerated flowering in the two Arabidopsis lines expressing HIF$_{ODD}$–PIF4. **E.** Leaf number measured when the floral stem reached 1 cm in length. **F.** Hypocotyl length of plants expressing *HIF$_{ODD}$-PIF4* and SLY1-2-VHL in the presence or absence of the PHD3 enzyme. Statistical significance, as determined using two-way ANOVA followed by Tukey's post-test ($p \leq 0.05$) is indicated by letters. **G.** Phenotypes of three-

week-old Arabidopsis Col-0 and transgenic lines carrying *HIF$_{ODD}$-PIF4* with or without PHD3. **H.** Petiole length of plants expressing *HIF$_{ODD}$-PIF4* and SLY1-2-VHL in the presence or absence of the PHD3 enzyme. Statistical significance, as determined using two-way ANOVA followed by Tukey's post-test ($p \leq 0.05$) is indicated by letters.

Supplementary Table 1: List of plasmids used in this study

	Plasmid	Description
1	35S:TIR1$_{Fbox}$-VHL (small for protopalsts)	This study
2	35S:HIF-GAL4-AD-FLAG (small for protoplasts)	This study
3	35S:PHD3 (small for protoplasts)	[7]
4	prom4xUAS:FLUC (small for protoplasts)	[8]
5	35S:SLY1-VHL	This study
6	35S:SLY1-2-VHL	This study
7	35S:SLY1-10-VHL	This study
8	35S:GFP -SLY1-2-VHL	This study
9	pP16-NLS-VENUS-HIF$_{ODD}$	This study
10	p35S-PHD3-HygR	This study
11	35S:Ω:RLuc-UBQ-HIF$_{ODD}$-Fluc-HIF$_{ODD}$	This study
12	35S:ADH:RLuc-UBQ-HIF$_{ODD}$-Fluc-HIF$_{ODD}$	This study
13	35S:ADH:RLuc-UBQ- Fluc-HIF$_{ODD}$	This study
14	35S:Ω:FLuc-UBQ-nLuc-HIF$_{ODD}$	This study
15	35S:Ω:FLuc-UBQ-HIF$_{ODD}$nLucHIF$_{ODD}$	This study
16	35S:ADH:FLuc-UBQ-nLuc-HIF$_{ODD}$	This study
17	35S:ADH:FLuc-UBQ-HIF$_{ODD}$nLucHIF$_{ODD}$	This study
18	35S:PHD3(R205K)	This study
19	35S:PHD3(D137H)	This study
20	35S:PHD3(D137E)	This study
21	35S:PHD3(D315E)	This study
22	35S:PHD3(R383K)	This study
23	promRAP2.12:HIF$_{ODD}$-2xHA-Δ13RAP2.12/SLY1-2-VHL	This study
24	promRAP2.12RAP2.12-2xHA	This study
25	promPCO4:PHD3	This study
26	pUBQ10: HIF$_{ODD}$ -PIF4	This study
27	pSIC: HIF$_{ODD}$ -PIF4	This study
28	PGL3 PGK6 TkPluc	[9]
29	PGL4 RL	[9]
30	PCDNA3-HIF-1α	This study
31	PCDNA3-HIF-1α (PPAA)	This study
32	PCDNA3-RGS4$_{1-11}$-HIF-1α (PPAA)	This study
33	pF7pPCO4-PHD3	This study

Supplementary Table 2: List of oligonucleotides used in this study

Name	Sequence
3xHA_Fw	caccatggggcaacaaggatacccg
UBQ10_Rv	ctttatgtttttggcgtcttcaccacctcttaacctg
FLuc_Fw	caggttaagaggtggtgaagacgccaaaaacataaag
FLuc_Hif_Rv	tcaagatctaagttgaaaatcatcatccataggaatataaggagcaag catttcaagatcaagatcagtatccaatttggactttccgcccttctt ggcc
p16_Fw	gggaggcctaatggaaccatcttttg
p16_Rv	cccctagttttcagagcaggaagg
NcoI_pRAP_Fw	gggccatggcatgatggatatgaac
NcoI_pRAP_Rv	cccccatggggcggcgattcttgagg
ApaI_pOLE_Fw	ccgggcccgtatgtaggtatagtaac
CpoI_mCHERRY_Rv	ggggcggtccgtcacttgtgccccag
ApaI_At2S3_Fw	cgggcccggaggaaaccaaattaac
CpoI_t35S_Rv	ggtccgggtgatctgacgcctcg
PHD3Asp137His_Fw	atgttagacatgttcataatcctaatggagatgg
PHD3Asp137His_Rv	ccatctccattaggattatgaacatgtctaacat
PHD3Asp137Glu_Fw	atgttagacatgttgaaaatcctaatggagatgg
PHD3Asp137Glu_Rv	ccatctccattaggattttcaacatgtctaacat
PHD3Arg205Lys_Fw	ccttcttatgctactaaatatgctatgactgtt
PHD3Arg205Lys_Rv	aacagtcatagcatatttagtagcataagaagg
StuI_pAt4g24500_Fw	aggccttcttctccgacgactgtgag
SpeI_pAt4g24500_Fw	actagttgtgattcaactacaagatcaaaagg
At1G43800_F (SAD6)	ttggcaacccgcttctttcttacc
At1G43800_R (SAD6)	tttccctcagctcacgaacctg
At5g15120_F (PCO1)	attgggtggttgatgctccaatg
At5g15120_R (PCO1)	atgcatgttcccgccatcttc
gHIF1α-FW	tgagagttccttatgtgtgcagt
gHIF1α-RV	tcaaaacattgcgaccacct
gHIF2α-FW	caatgcctttgcaccatccc
gHIF2α-RV	agcaaagccaaacccaatgc
HIFfw1	accttgagatgctcgctcct
PIF4rv1	cgcctgttggtagagaggct

Supplementary Table 3. Reactions and reaction rates of mathematical model

	Reactions	Reaction Rates	Description
v_1	→ RAP2.12	k_1	RAP2.12 synthesis
v_2	RAP2.12 → null	$k_2 \cdot [RAP2.12]$	RAP2.12 degradation
v_3	RAP2.12 + O_2 + PCO4 → PCO4	$k_3 \cdot [PCO4] \cdot \frac{[O_2]}{Km_1+[O_2]} \cdot \frac{[RAP2.12]}{Km_2+[RAP2.12]}$	RAP2.12 degradation through oxidation by PCO4
v_4	RAP2.12 → mRNA + RAP2.12	$k_4 \cdot \frac{[RAP2.12]}{Km_3+[RAP2.12]}$	HRG mRNA production by RAP2.12
v_5	mRNA → null	$k_5 \cdot [mRNA]$	HRG mRNA decay
v_6	→ HIF_{ODD}-ΔRAP2.12	k_1	HIF_{ODD}-ΔRAP2.12 synthesis
v_7	HIF_{ODD}-ΔRAP2.12 → null	$k_2 \cdot [HIF_{ODD}\text{-}\Delta RAP2.12]$	HIF_{ODD}-ΔRAP2.12 degradation
v_8	HIF_{ODD}-ΔRAP2.12 + O_2 + PHD → PHD	$k_3 \cdot [PHD] \cdot \frac{[O_2]}{Km_1+[O_2]} \cdot \frac{[HIF_{ODD}-\Delta RAP2.12]}{Km_2+[HIF_{ODD}-\Delta RAP2.12]}$	HIF_{ODD}-ΔRAP2.12 degradation through hydroxylation by PHD
v_9	HIF_{ODD}-ΔRAP2.12 → mRNA + HIF_{ODD}-ΔRAP2.12	$k_4 \cdot \frac{[HIF_{ODD}-\Delta RAP2.12]}{Km_3+[HIF_{ODD}-\Delta RAP2.12]}$	HRG mRNA production by HIF_{ODD}-ΔRAP2.12

Supplementary Table 4. Ordinary differential equations of the kinetic model. The reaction rates v1– v9 are given in Supplementary Table 3.

	Left-hand Sides	Right-hand Sides	Simulated Initial Concentrations (nM)
RAP2.12/PCO4	d[RAP2.12]/dt	v1-v2-v3	0.9
model	d[RNA]/dt	v4-v5	0.0723
HIF_{ODD}-ΔRAP2.12/PHD	d[HIF_{ODD}-ΔRAP2.12]/dt	v6-v7-v8	5.8
model	d[RNA]/dt	v9-v5	0.4407

Supplementary Table 5. Parameter values used in the kinetic model. Concentrations and the Michaelis-Menten constants (Kms) are given in μM, except for Km values for O_2, which are given in %. Rate constants (k) are expressed in s^{-1}, except k_1 and k_4, which are reported in $nM{\cdot}s^{-1}$.

Parameters	Description	Values	References
k_1	RAP2.12/HIF_{ODD}-ΔRAP2.12 protein synthesis rate	0.005	[10,11]
k_2	RAP2.12/HIF_{ODD}-ΔRAP2.12 degradation rate	0.0002	[11]
k_3	Catalytic rate constant for PCO4-mediated oxidation of RAP2.12	26.5878	[5]Re-fitted
Km_1	Michaelis-Menten constant for O_2 as a substrate of PCO4	16.4217	[5]Re-fitted
Km_2	Michaelis-Menten constant for RAP2.12 as a substrate of PCO4	270.2505	[5]Re-fitted
PCO4/PHD	PCO4/PHD protein concentration	0.1	[12]
k_4	Activation rate constant for HRG expression by RAP2.12	0.0016	[13]
Km_3	Michaelis-Menten constant for HRG expression	0.05	Assumed
k_5	HRG mRNA decay rate	0.00038	[11]
k_6	Catalytic rate constant for PHD-mediated oxidation of HIF_{ODD}-ΔRAP2.12	0.1185	[6]Re-fitted
Km_4	Michaelis-Menten constant for O_2 as a substrate of PHD	27.3944	[6]Re-fitted
Km_5	Michaelis-Menten constant for HIF_{ODD}-ΔRAP2.12 as a substrate of PHD	7.8463	[6]Re-fitted

References for Supplementary Information

1. Tan, X. *et al.* Mechanism of auxin perception by the TIR1 ubiquitin ligase. *Nature* 446, (2007).

2. Stebbins, C. E., Kaelin, W. G. & Pavletich, N. P. Structure of the VHL-ElonginC-ElonginB Complex: Implications for VHL Tumor Suppressor Function. *Science (1979)* 284, 455–461 (1999).

3. Hon, W. C. *et al.* Structural basis for the recognition of hydroxyproline in HIF-1α by pVHL. *Nature 2002 417:6892* 417, 975–978 (2002).

4. Chowdhury, R. *et al.* Structural Basis for Binding of Hypoxia-Inducible Factor to the Oxygen-Sensing Prolyl Hydroxylases. *Structure* 17, 981–989 (2009).

5. White, M. D., Kamps, J. J. A. G., East, S., Taylor Kearney, L. J. & Flashman, E. The plant cysteine oxidases from Arabidopsis thaliana are kinetically tailored to act as oxygen sensors. *Journal of Biological Chemistry* 293, 11786–11795 (2018).

6. Tarhonskaya, H. *et al.* Investigating the contribution of the active site environment to the slow reaction of hypoxia-inducible factor prolyl hydroxylase domain 2 with oxygen. *Biochemical Journal* 463, (2014).

7. Iacopino, S. *et al.* A synthetic oxygen sensor for plants based on animal hypoxia signaling. *Plant Physiol* 179, (2019).

8. Weits, D. a *et al.* Plant cysteine oxidases control the oxygen-dependent branch of the N-end-rule pathway. *Nat Commun* 5, 3425 (2014).

9. Doran, D. M., Kulkarni-Datar, K., Cool, D. R. & Brown, T. L. Hypoxia Activates Constitutive Luciferase Reporter Constructs. *Biochimie* 93, 361 (2010).

10. Hargrove, J. L. Microcomputer-assisted kinetic modeling of mammalian gene expression. *The FASEB Journal* 7, (1993).

11. Nguyen, L. K. *et al.* A dynamic model of the hypoxia-inducible factor 1a (HIF-1a) network. *J Cell Sci* 128, (2015).

12. Schmierer, B., Novák, B. & Schofield, C. J. Hypoxia-dependent sequestration of an oxygen sensor by a widespread structural motif can shape the hypoxic response - a predictive kinetic model. *BMC Syst Biol* 4, (2010).

13. Kugel, J. F. & Goodrich, J. A. A kinetic model for the early steps of RNA synthesis by human RNA polymerase II. *Journal of Biological Chemistry* 275, 40483–40491 (2000).
