# Supplementary material for: Prolyl hydroxylase-dependent proteolysis enables the orthogonal hypoxia responses in plants
Source: Nat Commun. 2026 Apr 9;17:4834. doi: 10.1038/s41467-026-71366-3 (PMC13223302; doi:10.1038/s41467-026-71366-3)
Supplement: Supplementary file 2 — Description of Additional Supplementary Files [file 41467_2026_71366_MOESM2_ESM.pdf]

Description of Additional Supplementary Files

File Name: Supplementary Data 1

Description: Relative (log2FC) and absolute (FPKM) expression level of A. thaliana genes in wild type, erfVII and HIFODD-RAP2.12 (line #1) plants.

File Name: Supplementary Data 2

Description: Absolute (FPKM) expression level of 48 core hypoxia response genes of Arabibidopsis thaliana in wild type, erfVII and HIFODD-RAP2.12 (line #2) plants. These data are shown in Fig. 4E.

File Name: Supplementary Data 3

Description: Maps of plasmids used in the study.
