# Supplementary material for: Prolyl hydroxylase-dependent proteolysis enables the orthogonal hypoxia responses in plants
Source: Nat Commun. 2026 Apr 9;17:4834. doi: 10.1038/s41467-026-71366-3 (PMC13223302; doi:10.1038/s41467-026-71366-3)
Supplement: Supplementary file 5 — Reporting Summary [file 41467_2026_71366_MOESM5_ESM.pdf]

nature portfolio

Corresponding author(s): Beatrice Giuntoli
Francesco Licausi

Last updated by author(s): 04/01/2026

# Reporting Summary

Nature Portfolio wishes to improve the reproducibility of the work that we publish. This form provides structure for consistency and transparency in reporting. For further information on Nature Portfolio policies, see our Editorial Policies and the Editorial Policy Checklist.

## Statistics

For all statistical analyses, confirm that the following items are present in the figure legend, table legend, main text, or Methods section.

| n/a | Confirmed | |
|---|---|---|
| | ✗ | The exact sample size (*n*) for each experimental group/condition, given as a discrete number and unit of measurement |
| ✗ | | A statement on whether measurements were taken from distinct samples or whether the same sample was measured repeatedly |
| | ✗ | The statistical test(s) used AND whether they are one- or two-sided<br>*Only common tests should be described solely by name; describe more complex techniques in the Methods section.* |
| ✗ | | A description of all covariates tested |
| | ✗ | A description of any assumptions or corrections, such as tests of normality and adjustment for multiple comparisons |
| | ✗ | A full description of the statistical parameters including central tendency (e.g. means) or other basic estimates (e.g. regression coefficient) AND variation (e.g. standard deviation) or associated estimates of uncertainty (e.g. confidence intervals) |
| | ✗ | For null hypothesis testing, the test statistic (e.g. *F*, *t*, *r*) with confidence intervals, effect sizes, degrees of freedom and *P* value noted<br>*Give P values as exact values whenever suitable.* |
| ✗ | | For Bayesian analysis, information on the choice of priors and Markov chain Monte Carlo settings |
| ✗ | | For hierarchical and complex designs, identification of the appropriate level for tests and full reporting of outcomes |
| ✗ | | Estimates of effect sizes (e.g. Cohen's *d*, Pearson's *r*), indicating how they were calculated |

*Our web collection on statistics for biologists contains articles on many of the points above.*

## Software and code

Policy information about availability of computer code

| | |
|---|---|
| Data collection | qPCR data were collected using QuantStudio 7 Pro, western blot images were captured using iBright Analysis Software, confocal images were acquired with software developed for the Zeiss LSM800 confocal microscope. |
| Data analysis | Data analysis and statistics was calculated using R or GraphPad Prism 9 for Windows 10. |

For manuscripts utilizing custom algorithms or software that are central to the research but not yet described in published literature, software must be made available to editors and reviewers. We strongly encourage code deposition in a community repository (e.g. GitHub). See the Nature Portfolio guidelines for submitting code & software for further information.

## Data

Policy information about availability of data

All manuscripts must include a data availability statement. This statement should provide the following information, where applicable:

- Accession codes, unique identifiers, or web links for publicly available datasets
- A description of any restrictions on data availability
- For clinical datasets or third party data, please ensure that the statement adheres to our policy

Alla numerical data are provided as a supplementary file. RNA-seq data are available at https://www.ncbi.nlm.nih.gov/geo/query/acc.cgi?acc=GSE315308

## Research involving human participants, their data, or biological material

Policy information about studies with human participants or human data. See also policy information about sex, gender (identity/presentation), and sexual orientation and race, ethnicity and racism.

| | |
|---|---|
| Reporting on sex and gender | N/A |
| Reporting on race, ethnicity, or other socially relevant groupings | N/A |
| Population characteristics | N/A |
| Recruitment | N/A |
| Ethics oversight | N/A |

Note that full information on the approval of the study protocol must also be provided in the manuscript.

# Field-specific reporting

Please select the one below that is the best fit for your research. If you are not sure, read the appropriate sections before making your selection.

- [x] Life sciences
- [ ] Behavioural & social sciences
- [ ] Ecological, evolutionary & environmental sciences

For a reference copy of the document with all sections, see nature.com/documents/nr-reporting-summary-flat.pdf

# Life sciences study design

All studies must disclose on these points even when the disclosure is negative.

| | |
|---|---|
| Sample size | sample size was determined based on the amount of samples that was possible to processes/analyse in a single experiment. |
| Data exclusions | No data were excluded |
| Replication | Experiments were repeated twice or once with two independent transgenic lines. Experiments in figure 1E and F constitute independent repetitions of the same experiment, analysed with independent methods. Screenings were repeated only once. |
| Randomization | Allocation was not random to simplify handling. However, given the limited space occupied by experimental samples this is not expected to have affected the results. |
| Blinding | Blinding investigators increased chances of mixing-up the large amount of samples. Lack of binding did not affect the analysis of the results. |

# Reporting for specific materials, systems and methods

We require information from authors about some types of materials, experimental systems and methods used in many studies. Here, indicate whether each material, system or method listed is relevant to your study. If you are not sure if a list item applies to your research, read the appropriate section before selecting a response.

### Materials & experimental systems

| n/a | Involved in the study | |
|---|---|---|
| [ ] | [x] | Antibodies |
| [ ] | [x] | Eukaryotic cell lines |
| [ ] | [ ] | Palaeontology and archaeology |
| [ ] | [ ] | Animals and other organisms |
| [ ] | [ ] | Clinical data |
| [ ] | [ ] | Dual use research of concern |
| [ ] | [x] | Plants |

### Methods

| n/a | Involved in the study | |
|---|---|---|
| [ ] | [ ] | ChIP-seq |
| [ ] | [ ] | Flow cytometry |
| [ ] | [ ] | MRI-based neuroimaging |

## Antibodies

| | |
|---|---|
| Antibodies used | To detect the HA tag, membranes were probed with anti-HA primary antibody (Sigma-Aldrich; H3663) Membranes were then probed with HRP-conjugated anti-mouse secondary antibody (Sigma-Aldrich; 12-349) a<br>To detect the FLAG-tagged GAL4 activation domain, membranes were probed with the monoclonal HRP-conjugated, anti-FLAG antibody (A8592, Sigma-Aldrich).<br>To detect HIFODD-PIF4, a recombinant anti PIF4 antibody was used (AS163955, 2B Scientific) |

| | |
|---|---|
| | To detect HIF-1a and HIF-2a Mouse Anti-Human HIF-1α (610959, BD Transduction LaboratoriesTM) and anti-HIF-2α (mouse monoclonal, 190b) were used, respectively. HRP-conjugated secondary antibodies were purchased from DAKO. |
| Validation | Samples which did not express the target protein were included as controls in each blot. |

## Eukaryotic cell lines

Policy information about cell lines and Sex and Gender in Research

| | |
|---|---|
| Cell line source(s) | HKC clone 8, Homo sapiens, sex and age unspecified, HKC-8 cells were gifted by Prof Ser Peter Ratcliffe (10.1038/ncomms13183) |
| Authentication | None of the cell lines were authenticated |
| Mycoplasma contamination | Cells were not tested for mycoplasma contamination |
| Commonly misidentified lines (See ICLAC register) | *Name any commonly misidentified cell lines used in the study and provide a rationale for their use.* |

## Palaeontology and Archaeology

| | |
|---|---|
| Specimen provenance | NA |
| Specimen deposition | NA |
| Dating methods | NA |

☐ Tick this box to confirm that the raw and calibrated dates are available in the paper or in Supplementary Information.

| | |
|---|---|
| Ethics oversight | NA |

Note that full information on the approval of the study protocol must also be provided in the manuscript.

## Animals and other research organisms

Policy information about studies involving animals; ARRIVE guidelines recommended for reporting animal research, and Sex and Gender in Research

| | |
|---|---|
| Laboratory animals | NA |
| Wild animals | NA |
| Reporting on sex | NA |
| Field-collected samples | NA |
| Ethics oversight | NA |

Note that full information on the approval of the study protocol must also be provided in the manuscript.

## Clinical data

Policy information about clinical studies

All manuscripts should comply with the ICMJE guidelines for publication of clinical research and a completed CONSORT checklist must be included with all submissions.

| | |
|---|---|
| Clinical trial registration | NA |
| Study protocol | NA |
| Data collection | NA |
| Outcomes | NA |

## Dual use research of concern

Policy information about dual use research of concern

### Hazards

Could the accidental, deliberate or reckless misuse of agents or technologies generated in the work, or the application of information presented in the manuscript, pose a threat to:

| No | Yes | |
|---|---|---|
| ☒ | ☐ | Public health |
| ☒ | ☐ | National security |
| ☒ | ☐ | Crops and/or livestock |
| ☒ | ☐ | Ecosystems |
| ☒ | ☐ | Any other significant area |

## Experiments of concern

Does the work involve any of these experiments of concern:

| No | Yes | |
|---|---|---|
| ☒ | ☐ | Demonstrate how to render a vaccine ineffective |
| ☒ | ☐ | Confer resistance to therapeutically useful antibiotics or antiviral agents |
| ☒ | ☐ | Enhance the virulence of a pathogen or render a nonpathogen virulent |
| ☒ | ☐ | Increase transmissibility of a pathogen |
| ☒ | ☐ | Alter the host range of a pathogen |
| ☒ | ☐ | Enable evasion of diagnostic/detection modalities |
| ☒ | ☐ | Enable the weaponization of a biological agent or toxin |
| ☒ | ☐ | Any other potentially harmful combination of experiments and agents |

# Plants

| | |
|---|---|
| Seed stocks | Arabidopsis thaliana Columbia-0 seeds were originally obtained by the Nottingham Arabidopsis Stock Centre.<br>erfVII mutant was donated by Michael Holdsworth (University of Nottingham) |
| Novel plant genotypes | Col-0 plants transformed with dual luciferase ratiometric reporter using Agrobacterium-mediated transformation.<br>Col-0 plants transformed with fluorescent reporter using Agrobacterium-mediated transformation.<br>erfVII plants transformed with HIF-2xHA-RAP2.12 and RAP2.12-2xHA, under the control of RAP2.12 promoter, generated using Agrobacterium-mediated transformation.<br>Col-0 plants transformed with SLY-VHL, PHD3 and HIF-PIF4 using Agrobacterium-mediated transformation. |
| Authentication | The presence/expression of the transgene(s) were tested by PCR first and by qPCR, respectively. |

# ChIP-seq

## Data deposition

☐ Confirm that both raw and final processed data have been deposited in a public database such as GEO.

☐ Confirm that you have deposited or provided access to graph files (e.g. BED files) for the called peaks.

| | |
|---|---|
| Data access links<br>*May remain private before publication.* | NA |
| Files in database submission | NA |
| Genome browser session<br>(e.g. UCSC) | NA |

## Methodology

| | |
|---|---|
| Replicates | *Describe the experimental replicates, specifying number, type and replicate agreement.* |
| Sequencing depth | *Describe the sequencing depth for each experiment, providing the total number of reads, uniquely mapped reads, length of reads and whether they were paired- or single-end.* |
| Antibodies | *Describe the antibodies used for the ChIP-seq experiments; as applicable, provide supplier name, catalog number, clone name, and lot number.* |
| Peak calling parameters | *Specify the command line program and parameters used for read mapping and peak calling, including the ChIP, control and index files used.* |

Data quality | Describe the methods used to ensure data quality in full detail, including how many peaks are at FDR 5% and above 5-fold enrichment.

Software | Describe the software used to collect and analyze the ChIP-seq data. For custom code that has been deposited into a community repository, provide accession details.

# Flow Cytometry

## Plots

Confirm that:

- [ ] The axis labels state the marker and fluorochrome used (e.g. CD4-FITC).
- [ ] The axis scales are clearly visible. Include numbers along axes only for bottom left plot of group (a 'group' is an analysis of identical markers).
- [ ] All plots are contour plots with outliers or pseudocolor plots.
- [ ] A numerical value for number of cells or percentage (with statistics) is provided.

## Methodology

Sample preparation | Describe the sample preparation, detailing the biological source of the cells and any tissue processing steps used.

Instrument | Identify the instrument used for data collection, specifying make and model number.

Software | Describe the software used to collect and analyze the flow cytometry data. For custom code that has been deposited into a community repository, provide accession details.

Cell population abundance | Describe the abundance of the relevant cell populations within post-sort fractions, providing details on the purity of the samples and how it was determined.

Gating strategy | Describe the gating strategy used for all relevant experiments, specifying the preliminary FSC/SSC gates of the starting cell population, indicating where boundaries between "positive" and "negative" staining cell populations are defined.

- [ ] Tick this box to confirm that a figure exemplifying the gating strategy is provided in the Supplementary Information.

# Magnetic resonance imaging

## Experimental design

Design type | Indicate task or resting state; event-related or block design.

Design specifications | Specify the number of blocks, trials or experimental units per session and/or subject, and specify the length of each trial or block (if trials are blocked) and interval between trials.

Behavioral performance measures | State number and/or type of variables recorded (e.g. correct button press, response time) and what statistics were used to establish that the subjects were performing the task as expected (e.g. mean, range, and/or standard deviation across subjects).

## Acquisition

Imaging type(s) | Specify: functional, structural, diffusion, perfusion.

Field strength | Specify in Tesla

Sequence & imaging parameters | Specify the pulse sequence type (gradient echo, spin echo, etc.), imaging type (EPI, spiral, etc.), field of view, matrix size, slice thickness, orientation and TE/TR/flip angle.

Area of acquisition | State whether a whole brain scan was used OR define the area of acquisition, describing how the region was determined.

Diffusion MRI [ ] Used [ ] Not used

## Preprocessing

Preprocessing software | Provide detail on software version and revision number and on specific parameters (model/functions, brain extraction, segmentation, smoothing kernel size, etc.).

Normalization | If data were normalized/standardized, describe the approach(es): specify linear or non-linear and define image types used for transformation OR indicate that data were not normalized and explain rationale for lack of normalization.

Normalization template | Describe the template used for normalization/transformation, specifying subject space or group standardized space (e.g. original Talairach, MNI305, ICBM152) OR indicate that the data were not normalized.

Noise and artifact removal | *Describe your procedure(s) for artifact and structured noise removal, specifying motion parameters, tissue signals and physiological signals (heart rate, respiration).*

Volume censoring | *Define your software and/or method and criteria for volume censoring, and state the extent of such censoring.*

## Statistical modeling & inference

Model type and settings | *Specify type (mass univariate, multivariate, RSA, predictive, etc.) and describe essential details of the model at the first and second levels (e.g. fixed, random or mixed effects; drift or auto-correlation).*

Effect(s) tested | *Define precise effect in terms of the task or stimulus conditions instead of psychological concepts and indicate whether ANOVA or factorial designs were used.*

Specify type of analysis: ☐ Whole brain ☐ ROI-based ☐ Both

Statistic type for inference | *Specify voxel-wise or cluster-wise and report all relevant parameters for cluster-wise methods.*

(See Eklund et al. 2016)

Correction | *Describe the type of correction and how it is obtained for multiple comparisons (e.g. FWE, FDR, permutation or Monte Carlo).*

## Models & analysis

| n/a | Involved in the study |
|---|---|
| | ☒ Functional and/or effective connectivity |
| ☒ | Graph analysis |
| | ☒ Multivariate modeling or predictive analysis |

Functional and/or effective connectivity | Differential equation models as described in M&M and Supplementary information

Multivariate modeling and predictive analysis | As described in M&M and Supplementary information
