# Supplementary material for: Prolyl hydroxylase-dependent proteolysis enables the orthogonal hypoxia responses in plants
Source: Nat Commun. 2026 Apr 9;17:4834. doi: 10.1038/s41467-026-71366-3 (PMC13223302; doi:10.1038/s41467-026-71366-3)
Supplement: Supplementary file 7 — Source Data [file 41467_2026_71366_MOESM7_ESM.zip › Uncropped western blots 1225.pdf]

Figure 1f

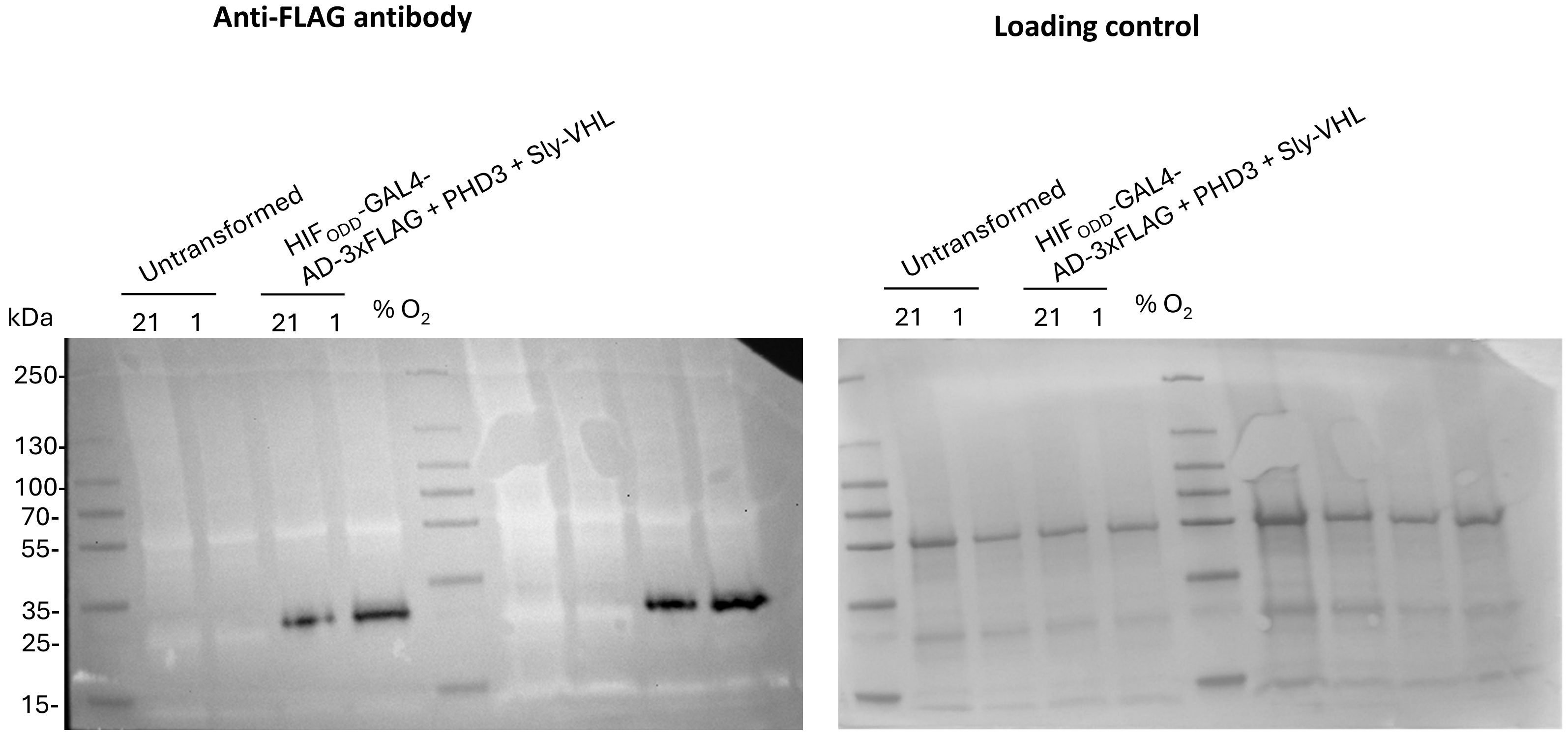

Anti-FLAG antibody
Loading control
Untransformed
HIF ODD-GAL4-AD-3xFLAG + PHD3 + Sly-VHL
kDa
21
1
21
1
% O2
250-
130-
100-
70-
55-
35-
25-
15-

Supplementary Figure 4d

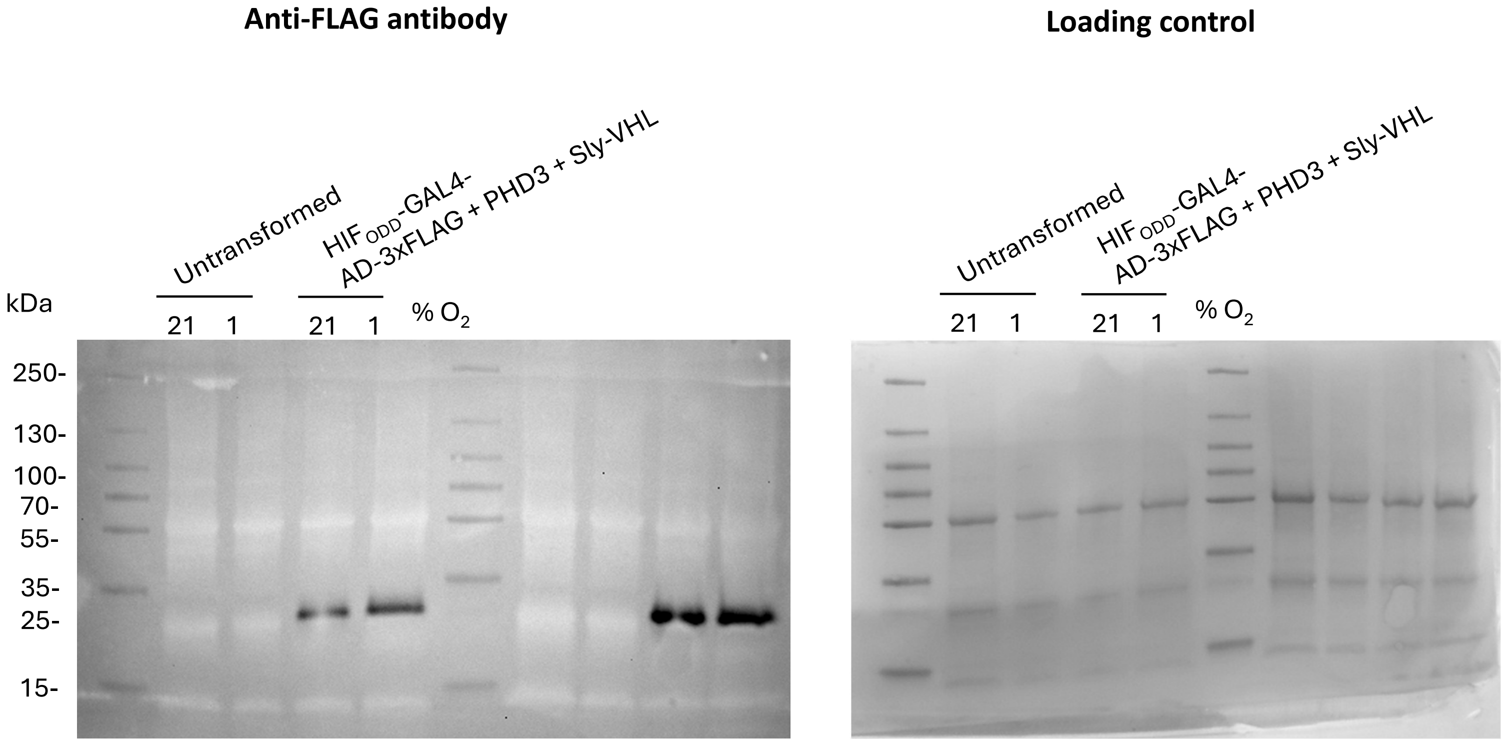

Figure 3b

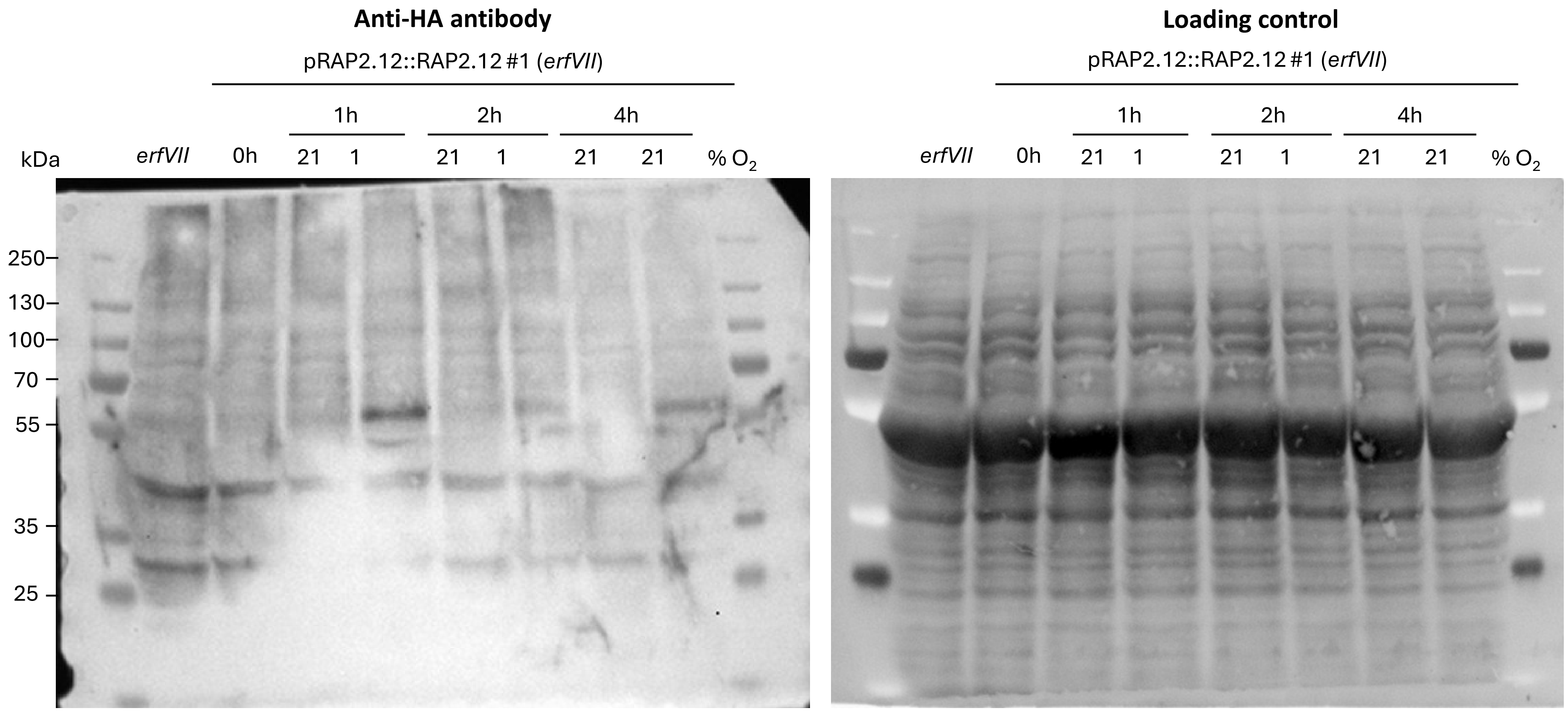

Anti-HA antibody
pRAP2.12::RAP2.12 #1 (erfVII)
1h
2h
4h
kDa
erfVII
0h
21
1
21
1
21
21
% O2
250
130
100
70
55
35
25
Loading control
pRAP2.12::RAP2.12 #1 (erfVII)
1h
2h
4h
erfVII
0h
21
1
21
1
21
21
% O2

Figure 3c

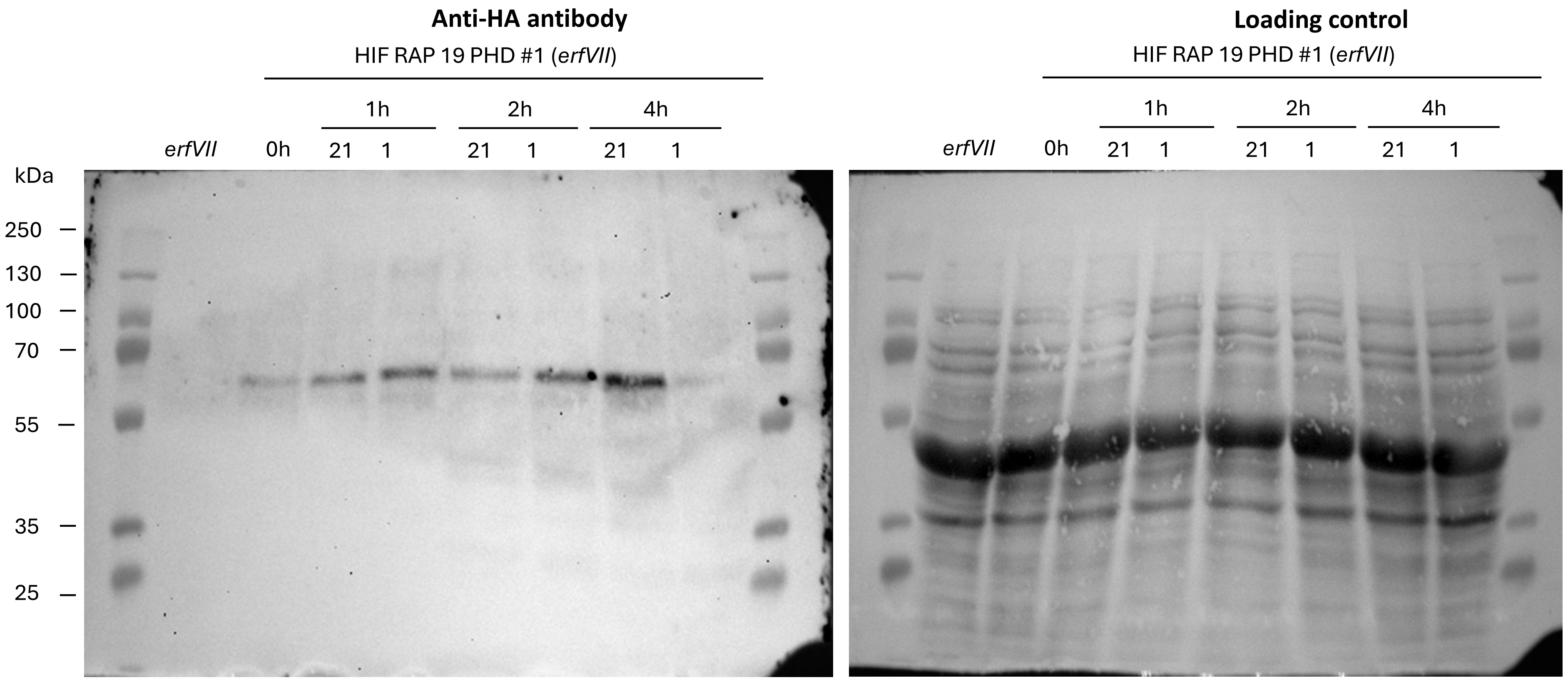
Anti-HA antibody
HIF RAP 19 PHD #1 (*erfVII*)
1h
2h
4h
erfVII
0h
21
1
21
1
21
1
kDa
250
130
100
70
55
35
25
Loading control
HIF RAP 19 PHD #1 (*erfVII*)
1h
2h
4h
erfVII
0h
21
1
21
1
21
1

Supplementary Figure 8a

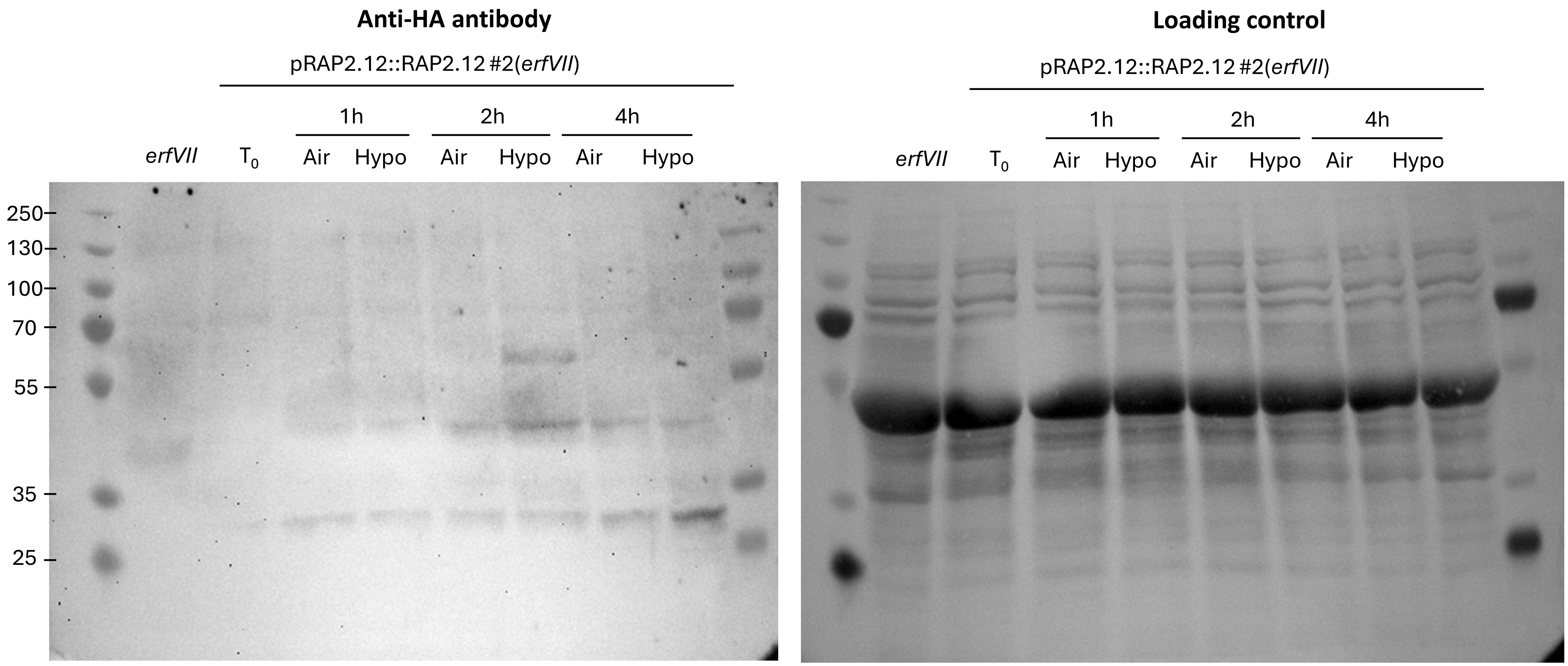

Supplementary Figure 8b

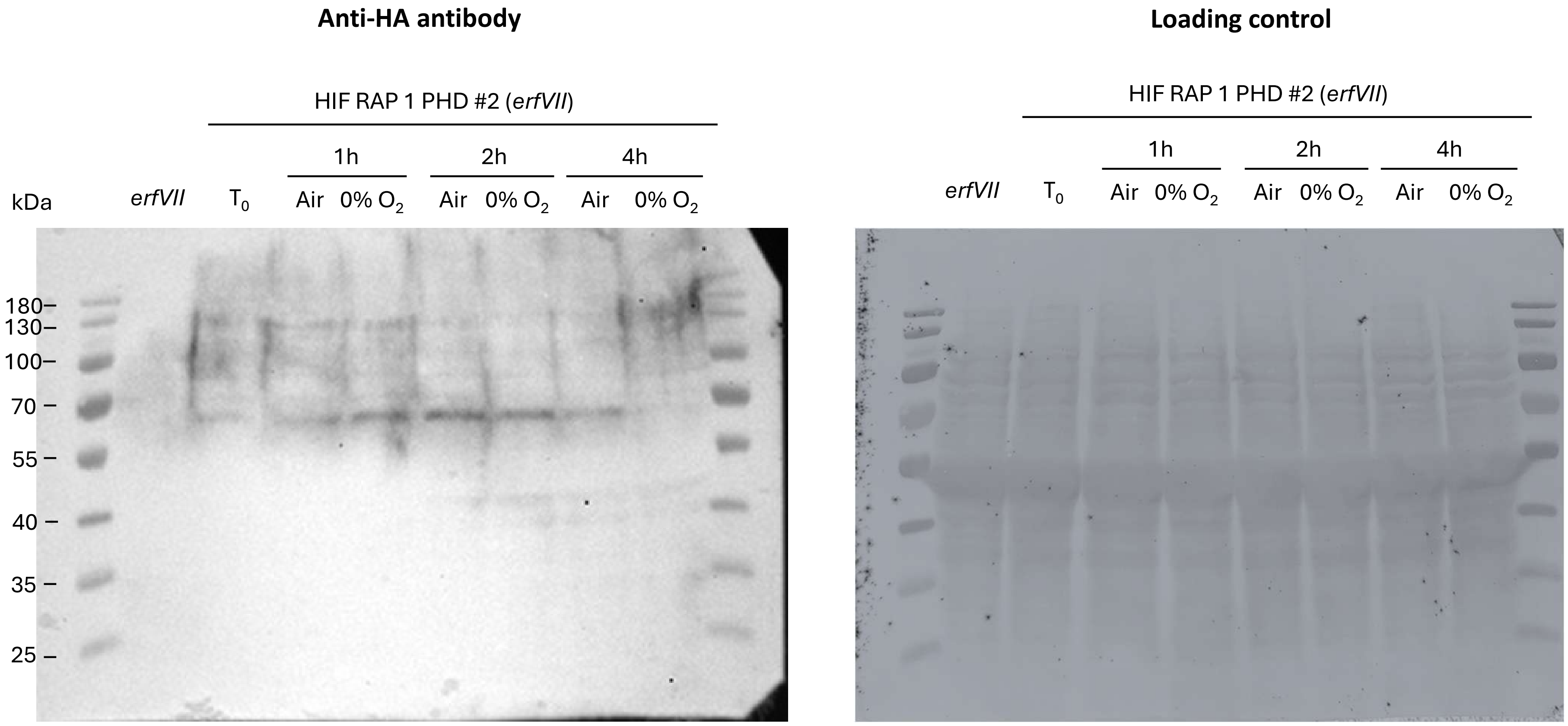

Supplementary Figure 8c

Anti-HA antibody

pRAP2.12:HIF_{ODD}- 2xHA-
ΔRAP2.12 (erfVII) #1

1h 2h 4h 6h

kDa

21 1 21 1 21 1 21 1

250-
130-
100-
70-
55-
35-
25-
15-

Loading control

pRAP2.12:HIF_{ODD}- 2xHA-
ΔRAP2.12 (erfVII) #1

1h 2h 4h 6h

21 1 21 1 21 1 21 1

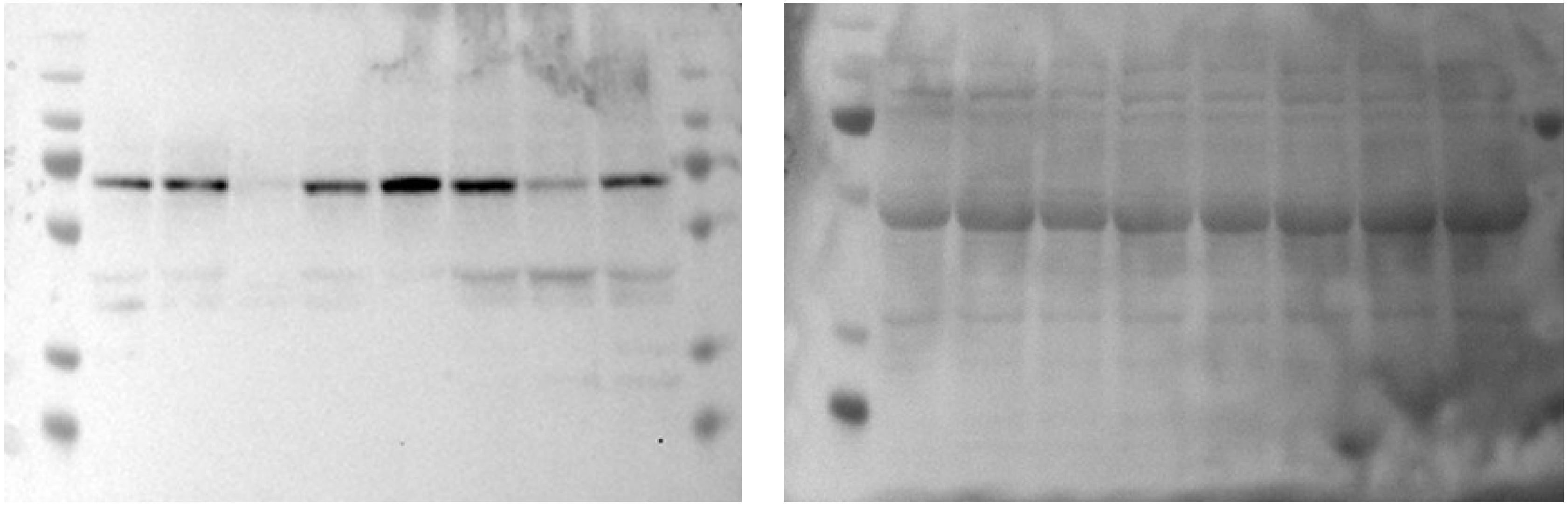

Figure 3d

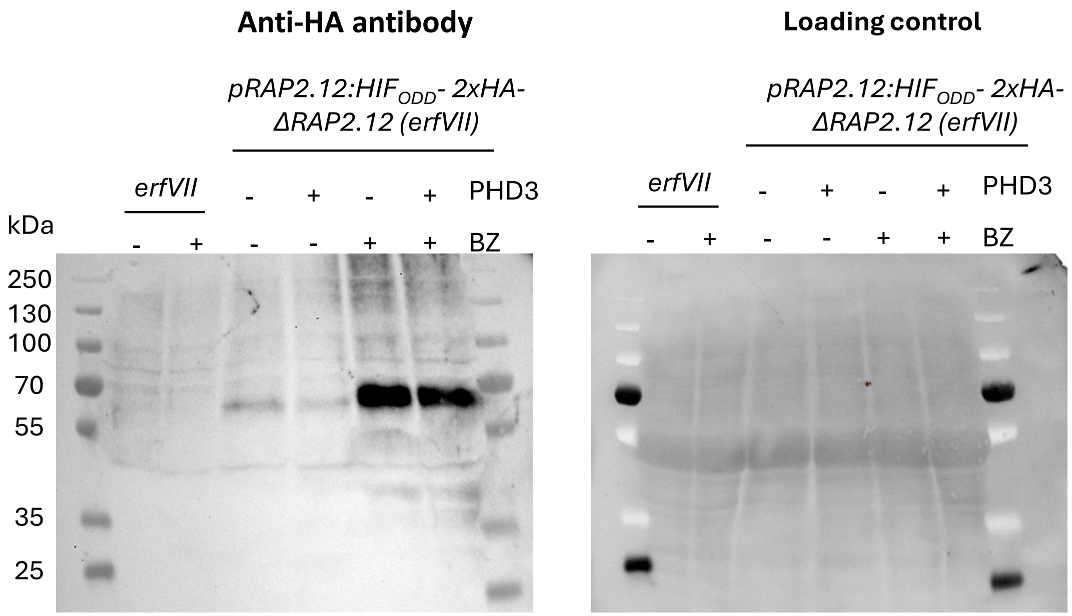
Anti-HA antibody
Loading control
pRAP2.12:HIF$_{ODD}$- 2xHA-ΔRAP2.12 (erfVII)
erfVII
PHD3
BZ
kDa
250
130
100
70
55
35
25

Figure 3f

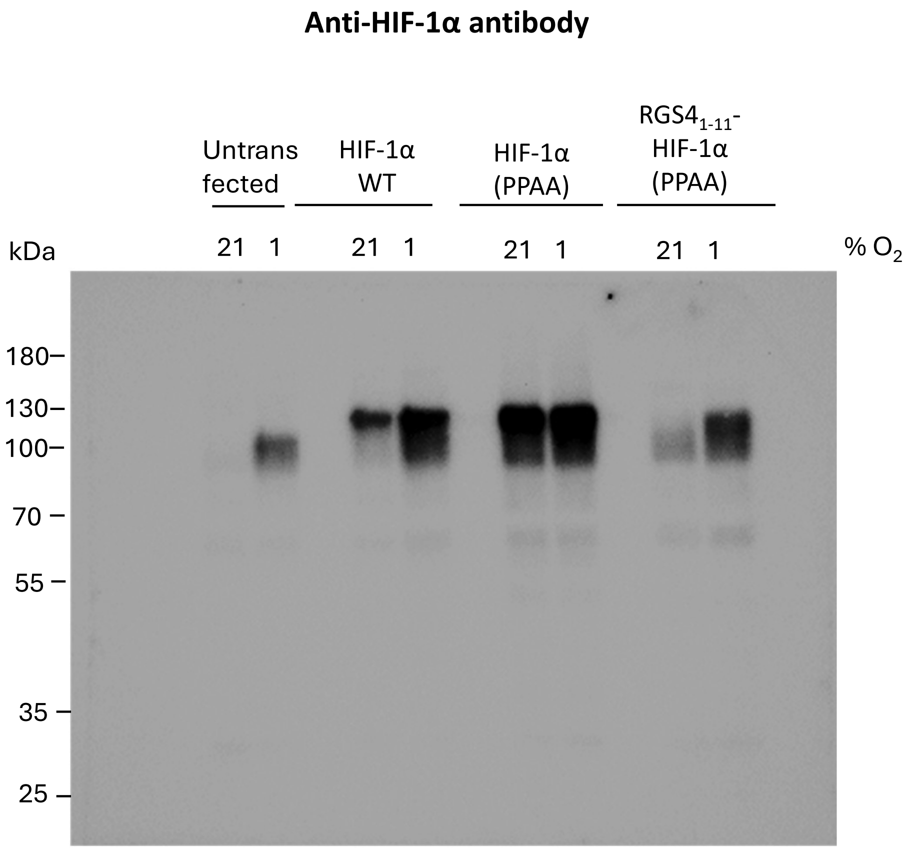

Anti-HIF-1α antibody
Untrans fected
HIF-1α WT
HIF-1α (PPAA)
RGS4₁₋₁₁-HIF-1α (PPAA)
kDa
21 1 21 1 21 1 21 1
% O2
180
130
100
70
55
35
25

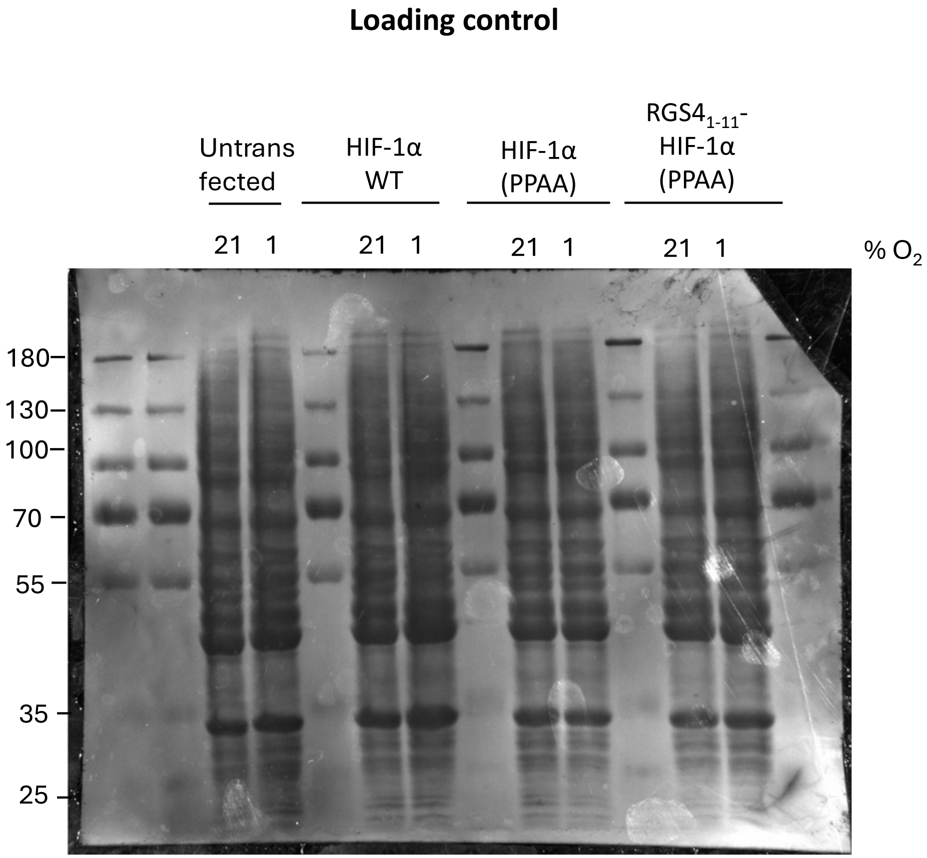

Loading control
Untrans fected
HIF-1α WT
HIF-1α (PPAA)
RGS4₁₋₁₁-HIF-1α (PPAA)
21 1 21 1 21 1 21 1
% O2
180
130
100
70
55
35
25

Supplementary Figure 8c

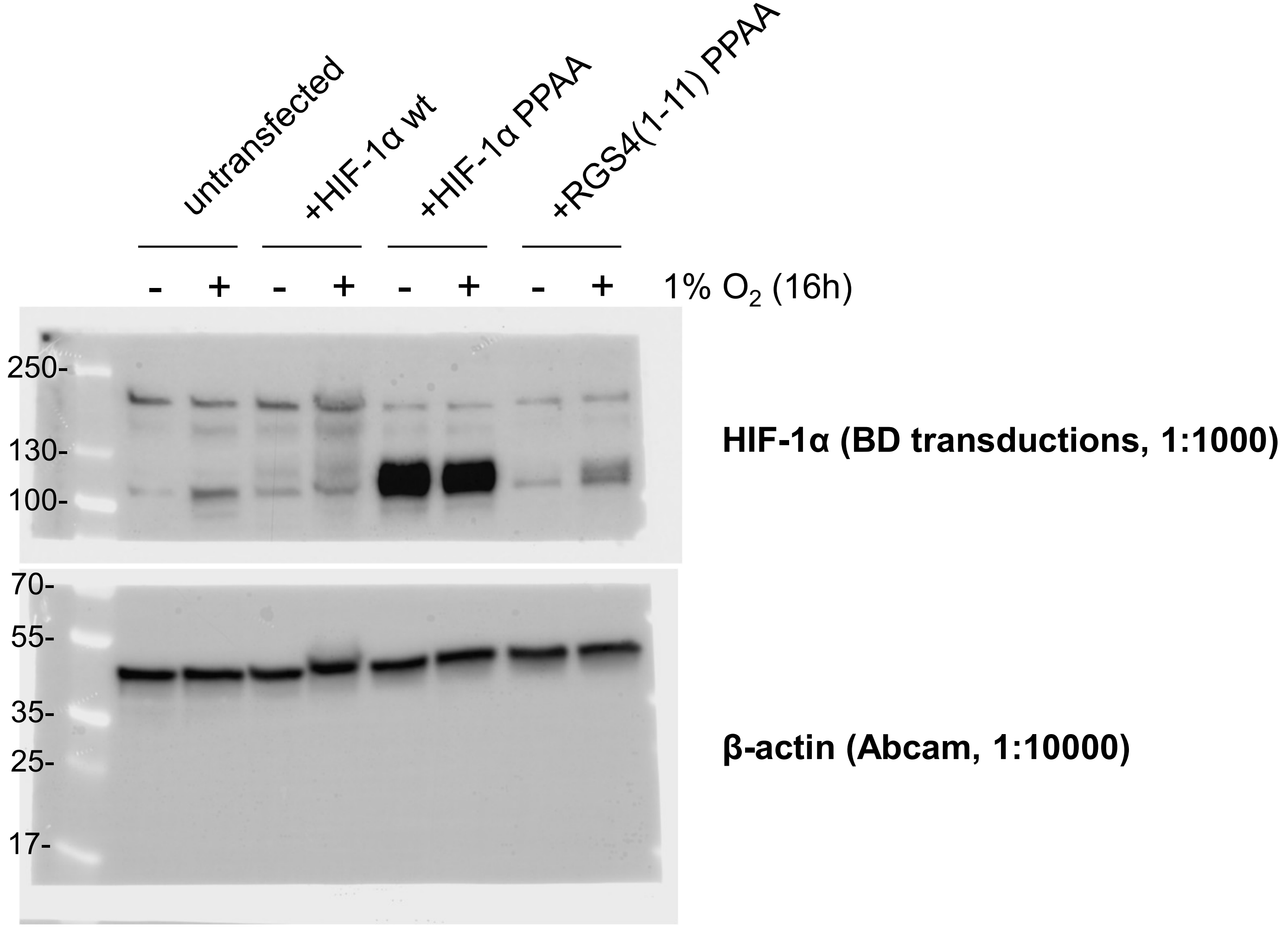

Figure 5b

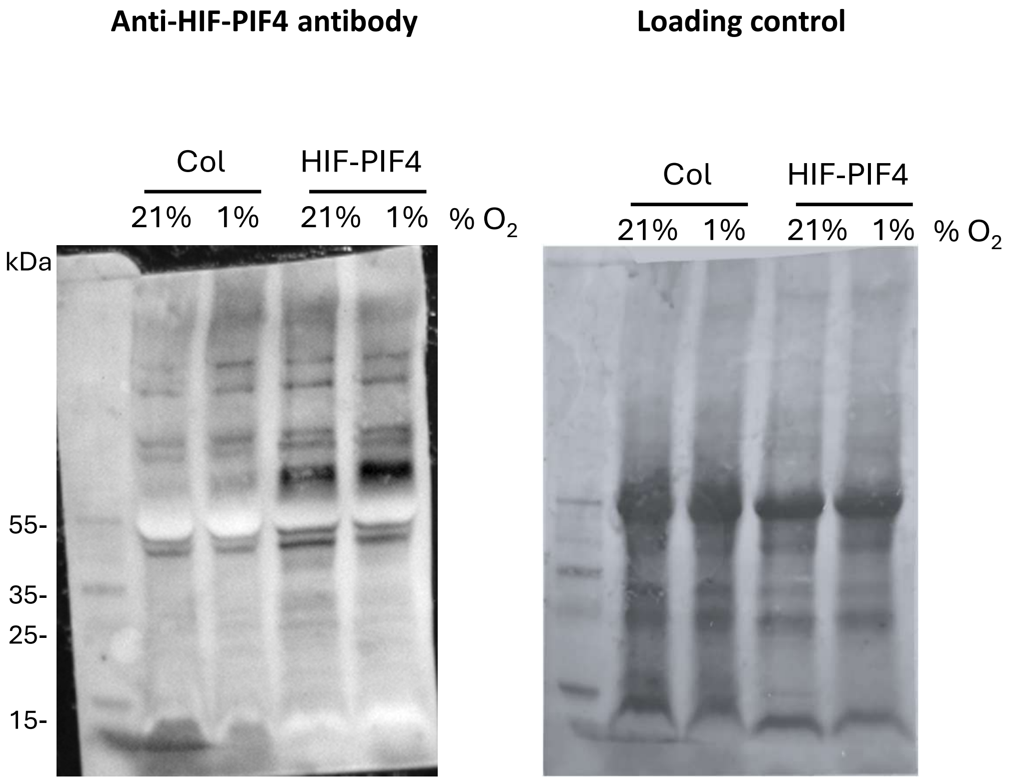

Anti-HIF-PIF4 antibody
Loading control
Col
HIF-PIF4
21%
1%
21%
1%
% O_2
kDa
55-
35-
25-
15-
Col
HIF-PIF4
21%
1%
21%
1%
% O_2

Figure 14b

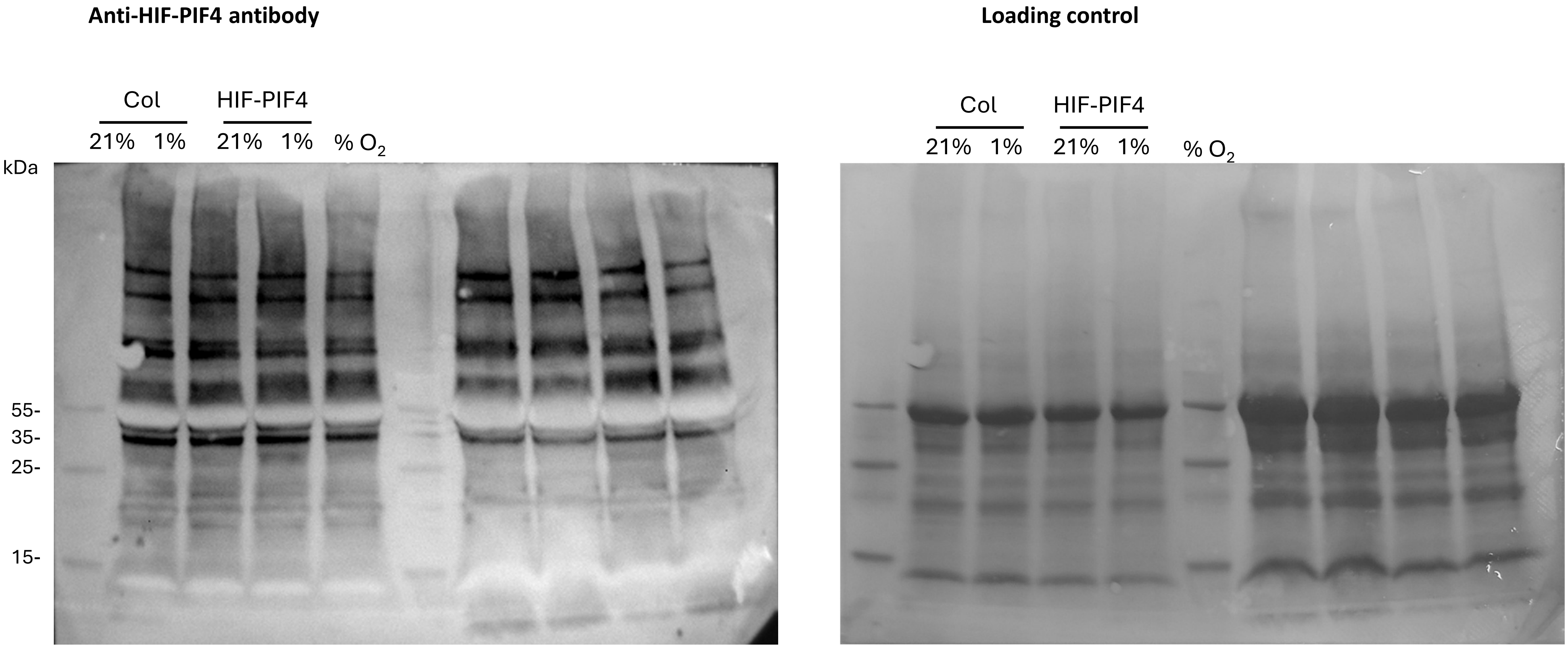

Anti-HIF-PIF4 antibody
Col
HIF-PIF4
21% 1% 21% 1% % O2
kDa
55-
35-
25-
15-
Loading control
Col
HIF-PIF4
21% 1% 21% 1% % O2
